# Erythrocytic α-synuclein contained in microvesicles regulates astrocytic glutamate homeostasis: a new perspective on Parkinson’s disease pathogenesis

**DOI:** 10.1186/s40478-020-00983-w

**Published:** 2020-07-08

**Authors:** Lifu Sheng, Tessandra Stewart, Dishun Yang, Eric Thorland, David Soltys, Patrick Aro, Tarek Khrisat, Zhiying Xie, Na Li, Zongran Liu, Chen Tian, Matthew Bercow, Junichi Matsumoto, Cyrus P. Zabetian, Elaine Peskind, Joseph F. Quinn, Min Shi, Jing Zhang

**Affiliations:** 1 Department of Pathology, University of Washington School of Medicine, Seattle, Washington USA; 2 Department of Pathology, Peking University Health Science Centre and Third Hospital, Beijing, China; 3 Geriatric Research, Education, and Clinical Center, Veterans Affairs Puget Sound Health Care System, Seattle, WA USA; 4 Department of Neurology, University of Washington School of Medicine, Seattle, WA USA; 5 Mental Illness Research Education and Clinical Center, VA Puget Sound Health Care System, Seattle, WA USA; 6 Department of Psychiatry and Behavioral Sciences, University of Washington School of Medicine, Seattle, WA USA; 7 Department of Neurology, Oregon Health and Science University, Portland, OR USA; 8 Department of Pathology, the First Affiliated Hospital and School of Medicine, Zhejiang University, Hangzhou, 310003 China

**Keywords:** Parkinson’s disease, Extracellular vesicles, Astrocytes, Blood-brain barrier, Alpha-synuclein, Glutamate

## Abstract

Parkinson’s disease is a neurodegenerative disorder characterized by the transmission and accumulation of toxic species of α-synuclein (α-syn). Extracellular vesicles (EVs) are believed to play a vital role in the spread of toxic α-syn species. Recently, peripheral α-syn pathology has been investigated, but little attention has been devoted to erythrocytes, which contain abundant α-syn. In this study, we first demonstrated that erythrocyte-derived EVs isolated from Parkinson’s disease patients carried elevated levels of oligomeric α-syn, compared to those from healthy controls. Moreover, human erythrocyte-derived EVs, when injected into peripheral blood in a mouse model of Parkinson’s disease, were found to readily cross the blood-brain barrier (BBB). These EVs accumulated in astrocyte endfeet, a component of the BBB, where they impaired glutamate uptake, likely via interaction between excitatory amino acid transporter 2 (EAAT2) and oligomeric α-syn. These data suggest that erythrocyte-derived EVs and the oligomeric α-syn carried in them may play critical roles in the progression or even initiation of Parkinson’s disease. Additionally, the mechanisms involved are attributable at least in part to dysfunction of astrocytes induced by these EVs. These observations provide new insight into the understanding of the mechanisms involved in Parkinson’s disease.

## Introduction

Parkinson’s disease is a neurodegenerative disorder characterized by both motor and nonmotor symptoms [40, 82]. Its major pathological hallmark is the accumulation of insoluble α-synuclein (α-syn) in deposits known as Lewy bodies. A role for α-syn in disease pathogenesis is further supported by the link between Parkinson’s disease and missense mutations or duplications/triplications of *SNCA*, the gene that encodes α-syn [1]. The protein is abundant in the brain, but is also found in remarkably high concentrations in the blood, particularly within the red blood cells (RBCs), i.e., erythrocytes [7, 43, 64, 81, 99]. In both the blood and the brain, it can be secreted into the extracellular space, and may be found either as free protein, or contained within extracellular vesicles (EVs), including exosomes and microvesicles. α-Syn-carrying EVs are believed to transmit Parkinson’s disease pathology [88], and have been found to cross the blood–brain barrier (BBB) in either direction [35, 53].

Many mechanisms have been implicated in the complex processes by which Parkinson’s disease arises. Recently, increasing attention has been paid to the role of astrocytes. One potential link may be glutamate homeostasis, a process that is under astrocytic control, and which has profound implications for neuronal survival. Astrocytic dysfunction resulting in reduced glutamate uptake, which has been reported in Parkinson’s disease, leads to abnormal levels of glutamate in the extracellular space, and subsequent neuronal excitotoxicity and neurodegeneration [9, 14]. Excitatory amino acid transporter 2 (EAAT2), an astrocyte-specific glutamate transporter, has been proposed to contribute to multiple neurodegenerative disorders [31, 45, 54, 95]. Astrocytes also play a major role in communication between the cells of the BBB and neurons, and BBB dysfunction is well-known to accompany Parkinson’s disease and other neurodegenerative diseases [23, 29, 42, 91]. Currently the links between astrocyte dysfunction and pathological α-syn are not entirely clear, and although astrocytes express much less α-syn than neurons [56], they can contain α-syn-positive inclusions in Parkinson’s disease [12], including in their processes [67, 87]. However, the source(s) of this astrocytic α-syn is not well-understood.

More recently, it has been hypothesized that transmission of α-syn pathology from the periphery to the brain could contribute to disease progression, and Parkinson’s disease might even originate outside of the central nervous system (CNS) [10, 11, 51, 71]. Our recent study showed that α-syn-containing EVs released by RBCs (RBC-EVs) could enter the brain in wild type (WT) mice, especially under conditions of BBB disruption induced by lipopolysaccharide (LPS) [53]. More interestingly, these RBC-EVs collected from plasma of Parkinson’s disease patients were found to induce inflammatory changes in brain microglia [53], suggesting that systemic spreading may also be a critical pathway of the periphery-to-CNS transmission of Parkinson’s disease pathology. Given the close proximity of astrocyte endfeet to the cerebral microvessels [23, 92], it is possible that α-syn or α-syn-carrying EVs could enter astrocytes (potentially among the first brain cells encountered by peripheral α-syn or α-syn-carrying EVs) from the peripheral blood. Thus, the connections between α-syn, including peripheral sources, and alterations in astrocyte function in Parkinson’s disease, deserve further study, particularly given the implication that astrocytes might mediate early responses to abnormalities of the BBB.

In this study, we further characterize RBC-EVs and explore their role in the progression of Parkinson’s disease pathology via astrocytes. We show that RBC-EVs can carry oligomeric species of α-syn into the CNS in a Parkinson’s disease mouse model [30], and that RBC-EVs from Parkinson’s disease subjects contain higher levels of oligomeric α-syn compared to healthy control subjects. Long-term injection of RBC-EVs facilitated abnormal α-syn deposition in brain. These EVs are likely first taken up by the astrocytic endfeet, leading to astrocytic dysfunction by reducing glutamate uptake via an interaction between EAAT2 and oligomeric α-syn. Our results demonstrate a potential novel mechanism for astrocyte-dependent Parkinson’s disease pathogenesis via peripheral abnormalities, implicating astrocyte interactions at the BBB as an early step in possible disease-related mechanisms induced by peripheral α-syn.

## Results

### Mutant α-syn-induced stress increases BBB permeability

BBB dysfunction has been reported as an early event during Parkinson’s disease pathogenesis [29], and has been observed in transgenic mice expressing human mutant A53T α-syn (A53T mice) [30]. Our previous study demonstrated that α-syn-carrying RBC-EVs enter the brain in WT mice under conditions of LPS-induced BBB disruption [53]. Therefore, we first characterized the contents and infiltration of RBC-EVs to the CNS in A53T mice, in which BBB function is compromised secondary to α-syn-driven pathology.

RBC-EVs carrying RBC marker CD235a [57, 61] were obtained from human plasma using an immunocapture protocol [79, 80]. Using Nanoparticle Tracking Analysis (NTA), we confirmed that the anti-CD235a immunocaptured RBC-EVs from human control and Parkinson’s disease plasma displayed a similar size distribution as those obtained from cultured RBCs in our previous study [53] (Supplemental Figure 1a-c). The specificity of the EV isolation was confirmed using non-specific mouse IgG capture as a negative control (Supplemental Figure 1c). CD235a was enriched in the CD235a-targeting, but not in the control IgG preparations (Supplemental Figure 1d). Captured RBC-EVs showed typical EV structures when imaged by CryoEM (Supplemental Figure 1f).

**Fig. 1:**
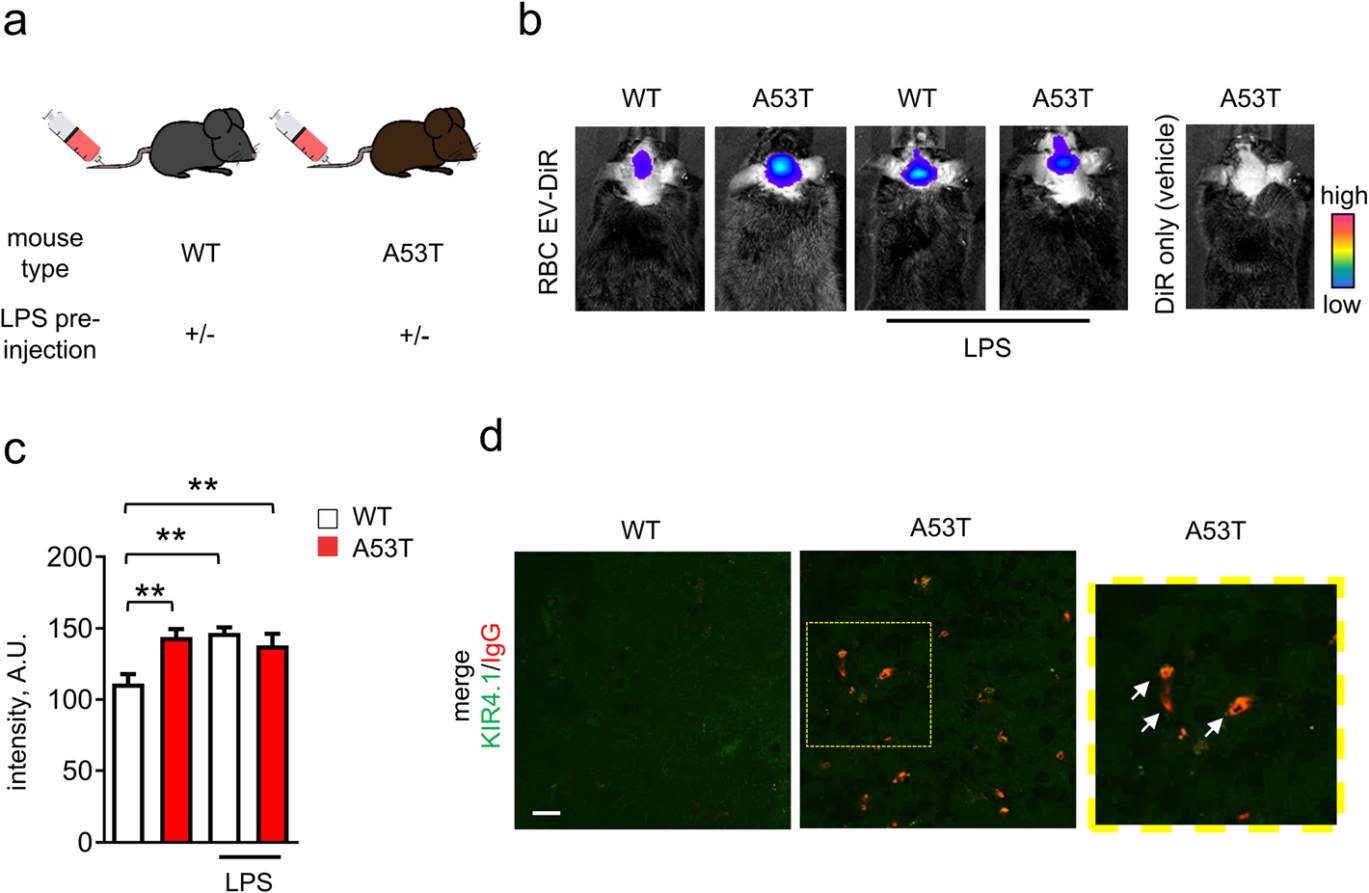
RBC-EVs can cross BBB in A53T mice. **a** Schematic representation showing the injection strategy. Briefly, RBC-EVs derived from control subjects were injected into WT or A53T mice in the presence or absence of LPS pre-administration. **b-c** Representative images and quantification of fluorescence signal of DiR-labeled RBC-EVs measured 3 h after injection. (*n* = 5 for WT injected with DiR-labeled RBC-EVs, *n* = 3 for other groups; means + S.E.M; **p < 0.001 by ordinary Two-way ANOVA test and Sidak’s multiple comparisons post-test). **d** Representative images of mouse brain slice from WT or A53T mouse (striatum) labeled with antibodies against mouse serum IgG and Kir4.1. Note that IgG signal can be only detected in A53T mice, and is co-localized with Kir4.1 labeled area

Isolated RBC-EVs were labelled with a fluorescent lipid dye (DiR), and injected i.v. into 3-month-old WT and A53T mice, in the presence or absence of a BBB-disrupting LPS pre-administration (Fig. 1a). Live animal imaging showed that fluorescence intensity of DiR-labeled RBC-EVs (DiR-EVs) in the brain of A53T mice was ~ 30% higher when compared with WT mice (Fig. 1b and c) in the absence of LPS pre-treatment. Of note, LPS-treated WT and A53T mice showed similar fluorescence intensity when compared to untreated A53T mice, suggesting that LPS cannot further promote the crossing of RBC-EVs from blood to CNS in A53T mice (Fig. 1b and c). No fluorescence was detected in A53T mice injected with fluorescent dye only (no EVs) (Fig. 1b).

These results suggest broad dysfunction of the BBB in A53T mice at a relatively early stage in their α-syn-related pathology [30]. To confirm this and explore the involvement of astrocytes in BBB leakage, brain slices from perfused 4 month old A53T or WT mice were labeled with antibodies against IgG, to evaluate BBB disruption [70], and inwardly rectifying potassium channel Kir4.1, an abundant and functionally important protein that labels astrocytes, including their processes and endfeet [20, 60]. Fluorescent IgG signals were detected in A53T mice, and co-localized with Kir4.1, while no IgG signal was detected in WT mice (Fig. 1d), confirming disruption of the BBB in A53T mice at early stages, as previously reported [30], and that astrocytic endfeet are in close proximity to the BBB leakages.

### RBC-EVs from Parkinson’s disease patients more potently promote α-syn aggregation in mouse brain

Our previous studies have demonstrated that RBC-derived EVs contain α-syn [53], and that RBCs from Parkinson’s disease patients contain increased levels of α-syn species, including oligomeric α-syn, compared to heathy control subjects [94]. Moreover, it is known that α-syn-carrying EVs from CSF or brains of Parkinson’s disease patients can promote the formation of α-syn aggregates when introduced into mouse brains [88]. Therefore, we hypothesized that blood-borne RBC-EVs entering the brain as a result of BBB dysfunction, particularly those carrying pathological forms of α-syn, may exacerbate the formation of α-syn pathology in the brain.

**Fig. 2:**
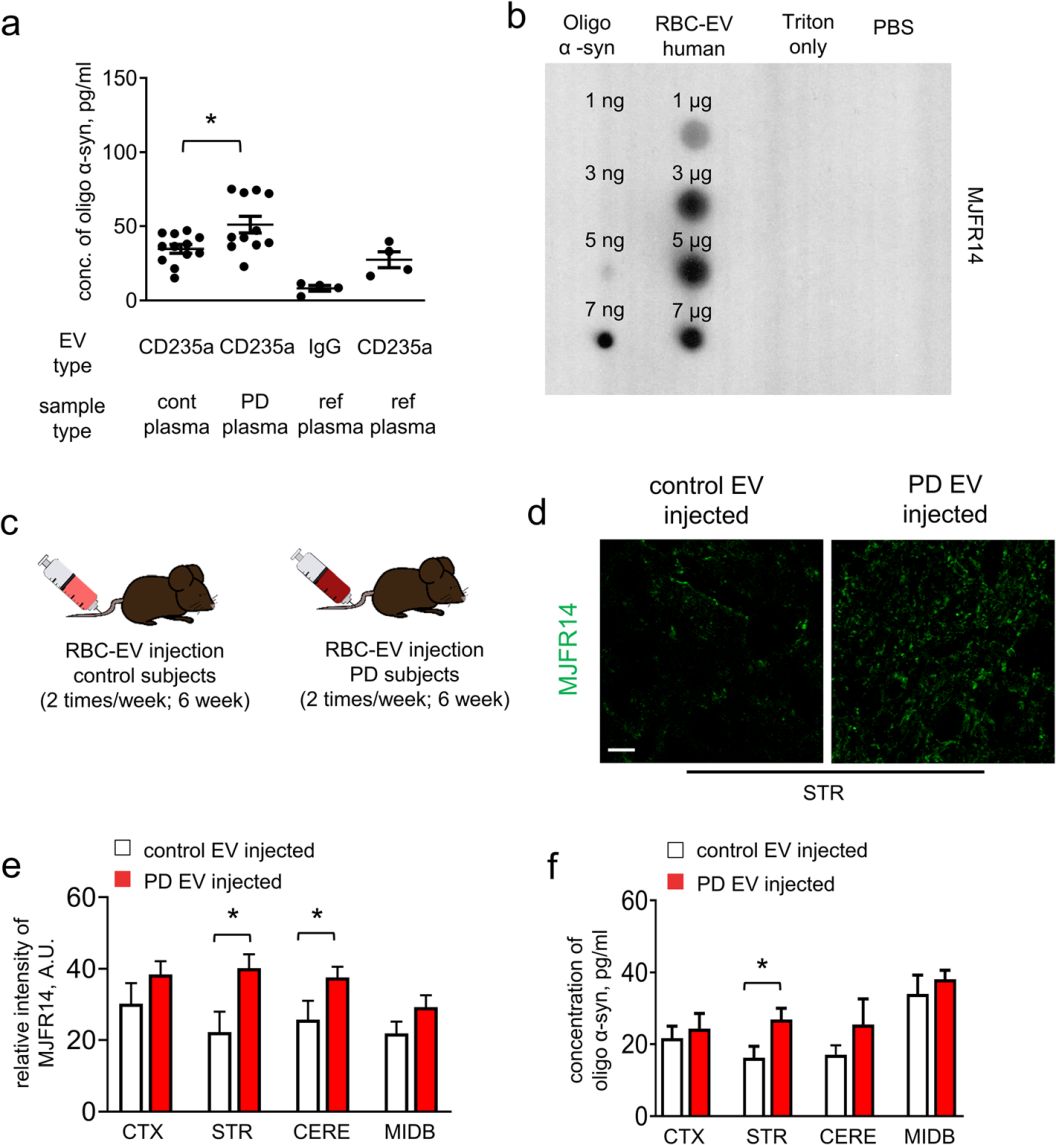
RBC-EVs contain oligomeric α-syn species and promote α-syn aggregation. **a** Levels of oligomeric α-syn in CD235a containing-EVs (RBC-EVs) in plasma were isolated by immuno-capture from healthy control subjects (*n* = 12 pools combined from 59 subjects), patients with PD (*n* = 11 pools combined from 109 subjects) and reference plasma samples (*n* = 4; pooled plasma generated from 30 healthy controls) or IgG captured EVs reference plasma samples (*n* = 4) measured by ECL immunoassays (means + S.E.M; *p < 0.05 by One-way ANOVA test). **b** Dot blot analysis of RBC-EVs isolated from control human subjects. Blots were probed with antibodies specifically against oligomeric α-syn, and oligomerized synthetic α-syn was used as a loading control. **c** Schematic representation showing the chronic injection strategy. RBC-EVs obtained from the plasma from human PD patients or healthy controls were injected 2 times/week i.v. into 3 month old A53T mice for 6 weeks. **d-e** After chronic RBC-EVs injection, mouse brain slices were immunofluorescently stained for oligomeric α-syn. Representative images of striatum (STR) (**d**) and quantification of fluorescence intensity of oligomeric α-syn in various brain regions (cortex (CTX), striatum (STR), cerebellum (CERE) and midbrain (MIDB) (**e**) are shown . Note that A53T mice injected with RBC-EVs derived from PD subjects showed increased levels of oligomeric α-syn fluorescence intensity compared to A53T mice injected with control RBC-EVs. (Scale bar, 10 μm, *n* = 5 independent animals were used in each group means + S.E.M; *p < 0.05 by One-way ANOVA test). **f** Mouse tissues from different brain regions were also homogenated after chronic RBC-EVs injection, and the levels of oligomeric α-syn in mouse brain homogenate of cortex (CTX), striatum (STR), midbrain (MIDB) and cerebellum (CERE) were measured by an ECL immunoassay (*n* = 5 independent animals were used in each group; means + S.E.M; *p < 0.05 by Student’s t-test *p < 0.05 compared to control-EVs injected group)

To test this notion, we measured oligomeric/fibrillar α-syn in RBC-EVs, from pooled plasma samples (see Supplemental Table 2) obtained from a cohort of 109 Parkinson’s disease and 59 healthy control subjects (See Supplemental Table 1 and Supplemental Table 2 for the clinical data summary). We isolated CD235a + EVs from plasma, and measured their oligomeric α-syn content using a recently developed and validated electrochemiluminescence (ECL)-based assay based on the Meso Scale Discovery (MSD) platform [94]. We observed that levels of oligomeric/fibrillar α-syn in Parkinson’s disease subjects were increased by ~ 30% when compared to control subjects (Fig. 2a). The control IgG-captured samples showed minimal oligomeric/fibrillar α-syn signal, suggesting that signal detected from Parkinson’s disease subjects was unlikely to be from contamination by free α-syn (Fig. 2a). Dot-blot analysis further confirmed that RBC-derived EVs contained oligomeric α-syn (Fig. 2b).

Having demonstrated elevated levels of oligomeric α-syn in RBC-EVs from Parkinson’s disease subjects, we next sought to investigate their effects on the brains of A53T mice. Parkinson’s disease or healthy control RBC-EVs were injected twice/week i.v. into 3-month-old A53T mice for 6 weeks (Fig. 2c). Immunofluorescence analysis was conducted to observe the progression of pathologic α-syn in the brains. Fluorescence intensity of oligomeric species of α-syn was increased by ~ 30% in striatum and ~ 20% in midbrain of mice injected with Parkinson’s disease compared to control RBC-EVs (Fig. 2d-e). Biochemical analysis of brain homogenate using the MSD assay showed increased oligomeric α-syn in striatum (~ 30%), with a trend toward increase in midbrain from the Parkinson’s disease vs control RBC-EV injected mice, and no significant effects in cortex and cerebellum (Fig. 2f). These results suggest that chronic exposure to oligomeric α-syn containing RBC-EVs may contribute to the accumulation of pathological α-syn in the brain in this Parkinson’s disease model.

### Astrocytes internalize RBC-EVs from the periphery and accumulate oligomeric α-syn in their processes

**Fig. 3:**
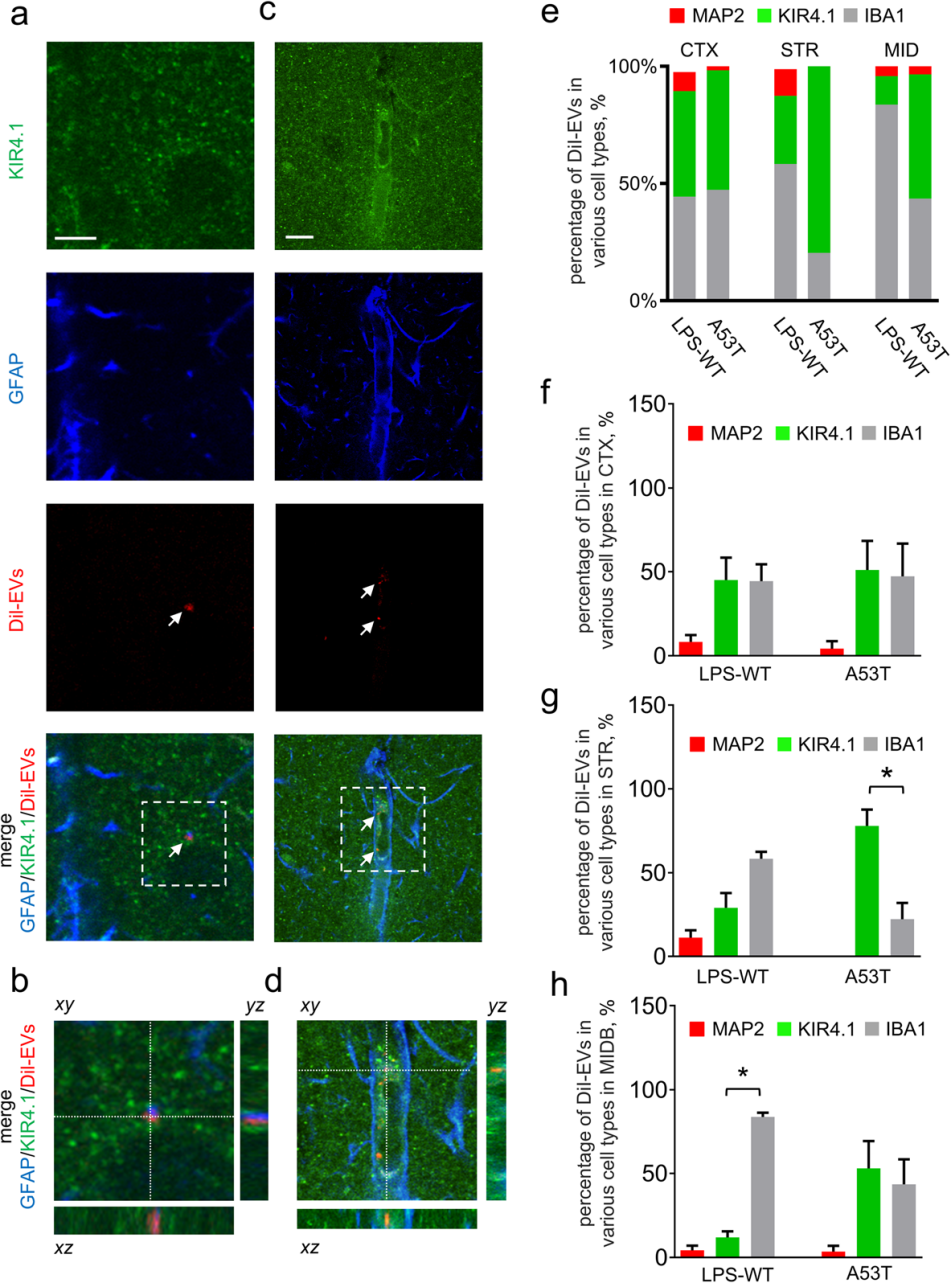
Astrocytes can take up RBC-EVs preferentially at endfeet in vivo. **a-d** Representative images of brain slices of striatum following peripheral injection of DiI-EVs and labeling with GFAP (marker for astrocytes) and Kir4.1 (marker for astrocytic endfeet). Note that DiI-EV co-localized with GFAP (**a-b**) and Kir4.1 (**c-d**) (Scale bar, 10 μm). **e** Analysis of the percentage of DiI-EVs colocalizing with MAP 2, Kir4.1 or IBA1 labeled cells in brain slices of LPS pre-administered WT mice or A53T mice (*n* = 5 for LPS pre-administered WT mice; *n* = 4 for A53T mice). **f-h** Graphs show analysis of the percentage of DiI-EVs colocalizing with MAP 2 (marker for neurons), Kir4.1 (marker for astrocytic endfeet) or IBA1 (marker for microglia) labeled cells in cortex (CTX, (**f**)), striatum (STR, (**g**)) and midbrain (MIDB, (**h**)). Note that increased percentage of DiI-EVs were colocalized with Kir4.1 at striatum in A53T mice compared with the LPS pre-administered WT mice (g; means + S.E.M; *n* ≥ 4; **p < 0.01 by One-way ANOVA test)

We next considered the possible involvement of astrocytes in the pathway that RBC-EVs might take between entering the brain and provocation of brain α-syn pathology. Our previous study showed uptake of RBC-EVs in the brain primarily by microglia. However, these results were obtained under conditions of LPS-induced systemic inflammation, which might alter microglial behavior [53]. Moreover, a number of RBC-EVs in the brain parenchyma were difficult to classify as being within a cell type, because their signal did not co-localize with any of the commonly used cell-type markers utilized in that study (GFAP for astrocytes, Map 2 for neurons, and Iba-1 for microglia). Notably, some EVs were in close proximity to astrocytes, often appearing quite near or even surrounded by their processes, with or without the signal co-localizing with GFAP (Fig. 3a-d). In light of the finding that in Parkinson’s disease model mice astrocytic endfeet, which typically express little or no GFAP, rather than the cell bodies, where GFAP is more abundant [30, 86], showed a close proximity to the BBB leakages, we examined whether RBC-EVs might be taken up by the distal processes of astrocytes. Animals were injected i.v. with human RBC-EVs, then perfused after 3 h. We found that RBC-EVs co-localized with the processes of GFAP labeled cells and Kir4.1 labeled astrocytic endfeet, suggesting that astrocytes do indeed take up RBC-EVs primarily at the endfeet regions that are in close physical proximity to the blood vessels (Fig. 3b and d). We next co-labeled brain slices with antibodies against Kir4.1, IBA1 and MAP 2 to investigate the proportion of RBC-EVs taken up by astrocytes, microglia, and neurons, respectively, in various brain regions. In the A53T mice, ~ 80% of DiI-labeled EVs co-localized with the Kir4.1-labeled astrocytic endfeet in the striatum, while ~ 30% were co-localized with the Kir4.1-labeled astrocytic endfeet in the striatum of LPS-treated mice (Fig. 3e and g). We did not observe any DiI-labeled EVs co-localized with MAP 2-labeled neurons in A53T mice, while ~ 10% were co-localized with MAP 2 labeled neurons in LPS-treated WT mice (Fig. 3g). In midbrain, ~ 50% of DiI-labeled EVs were co-localized with the Kir4.1-labeled astrocytic endfeet in A53T mice, while ~ 20% were co-localized with the Kir4.1-labeled astrocytic endfeet in LPS-treated mice (Fig. 3e and h), in alignment with the brain regions showing α-syn aggregation following chronic RBC-EV injection. This data indicates that RBC-EVs can be transported into brain astrocyte processes, and their fate in different cell types depends on brain region and the mechanism by which the BBB is disrupted.

**Fig. 4:**
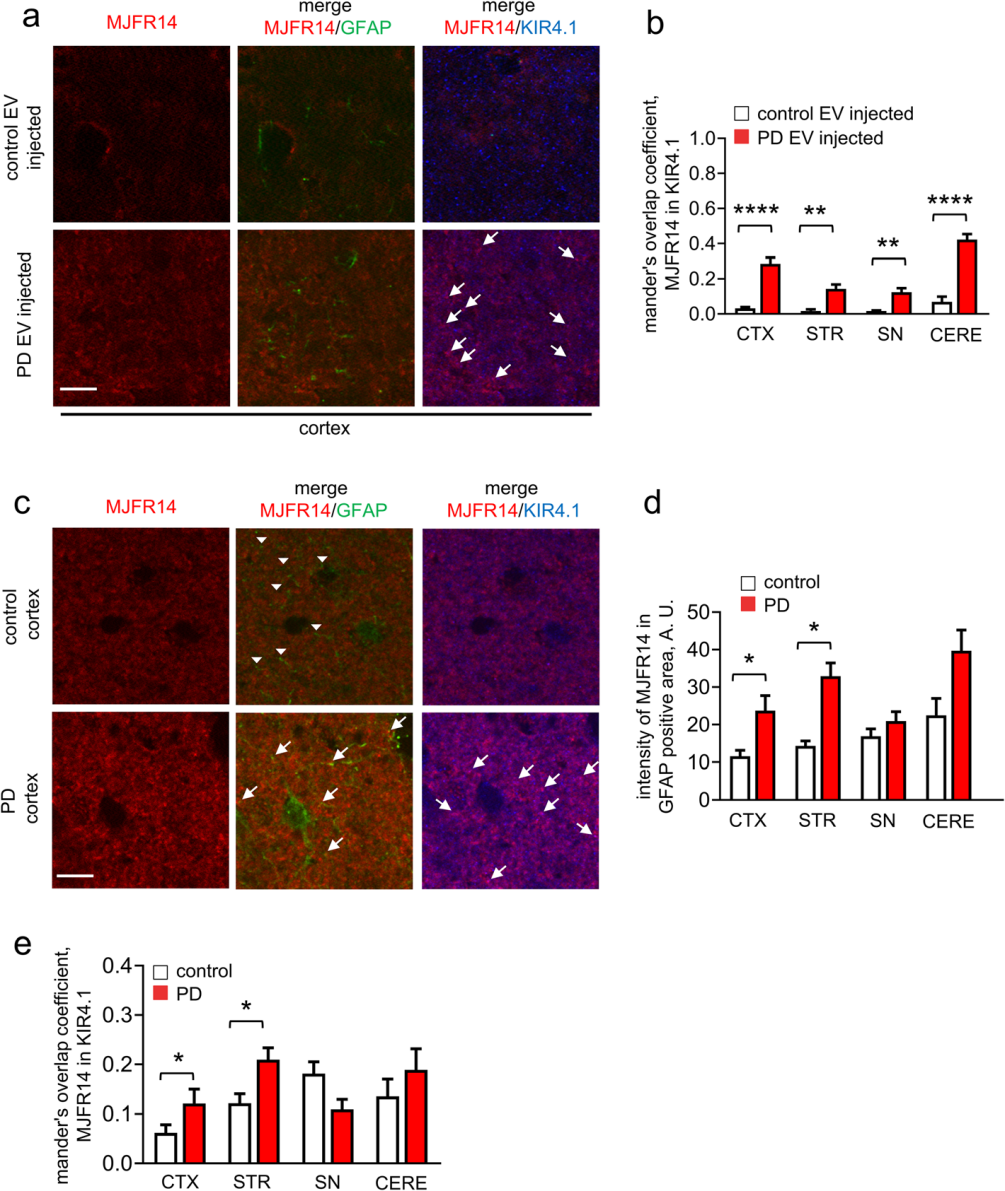
RBC-EVs facilitate α-syn aggregation in the processes of astrocytes. **a** Representative images of brain slices (cortex) following chronic peripheral injection of RBC-EVs derived from healthy control or PD subjects, co-labeled for oligomeric α-syn, GFAP (marker for astrocytes), and Kir4.1 (marker for astrocytic endfeet). Note the increased number of α-syn aggregations overlapping with Kir4.1 in brain slice from A53T mice injected PD RBC-EV (Scale bar, 10 μm). **b** Quantification analysis of oligomeric α-syn/ Kir4.1 co-localization (*n* ≥ 5 independent animals were used in each group means + S.E.M; *p < 0.05 by One-way ANOVA test). **c** Representative images of human postmortem tissues (cortex) co-labeled for oligomeric α-syn, GFAP, and Kir4.1. White arrow heads indicate GFAP-labeled astrocytic process without co-localization with oligomeric α-syn, while white arrows indicate GFAP-labeled or Kir4.1-labeled astrocytic process showing co-localization with oligomeric α-syn (Scale bar, 10 μm). **d-e** Quantification analysis of oligomeric α-syn/GFAP co-localization (**d**) and α-syn/ Kir4.1 co-localization (**e**) at cortex (CTX), striatum (STR), substantia nigra (SN) and cerebellum (CERE) (means + S.E.M; *n* = 5; **p < 0.01 by One-way ANOVA test)

We next analyzed whether chronic peripheral injection of RBC-EVs could result in elevated oligomeric α-syn in astrocytic endfeet, using the same six-week injection protocol. Immunolabeling showed that oligomeric α-syn often co-localized with the Kir4.1-labeled astrocytic endfeet (Fig. 4a). This was confirmed by quantification of oligomeric α-syn/ Kir4.1 co-localization, which was elevated in cortex, corpus striatum, midbrain and cerebellum of mice injected with Parkinson’s disease compared to control RBC-EVs (Fig. 4b). These results suggest that exposure to blood-borne α-syn elevates astrocytic α-syn primarily at the processes, and that RBC-EV-contained oligomeric α-syn likely promotes the formation of brain aggregates in astrocytes.

To corroborate this observation in human disease, we used brain tissue from 5 Parkinson’s disease and 5 neurologically normal control subjects (Supplemental Table 3) and immunostaining for oligomeric α-syn [94]. We found that oligomeric α-syn did not co-localize with GFAP-labeled astrocyte somata (Fig. 4c and Supplemental Figure 2). In agreement with our mouse experiment, we found an increase in oligomeric α-syn in GFAP+ astrocytic processes of Parkinson’s disease patients in cortex (~ 2 folds), striatum (~ 2 folds), and cerebellum (~ 2 folds) (Fig. 4c-d). However, no significant increase in oligomeric α-syn in astrocytes was observed in substantia nigra of Parkinson’s disease patients (Fig. 4c-d). We also observed that oligomeric α-syn often co-localized with Kir4.1-labeled astrocytic endfeet, suggesting that oligomeric α-syn in astrocytes may primarily reside in their processes. This was confirmed by quantification of oligomeric α-syn/Kir4.1 co-localization, which was elevated in cortex and corpus striatum of Parkinson’s disease subjects (Fig. 4c) compared to controls, further indicating that oligomeric α-syn aggregated at the astrocytic endfeet.

### RBC-EV-induced oligomeric α-syn pathology in astrocytes affects glutamate clearance via EAAT2

**Fig. 5:**
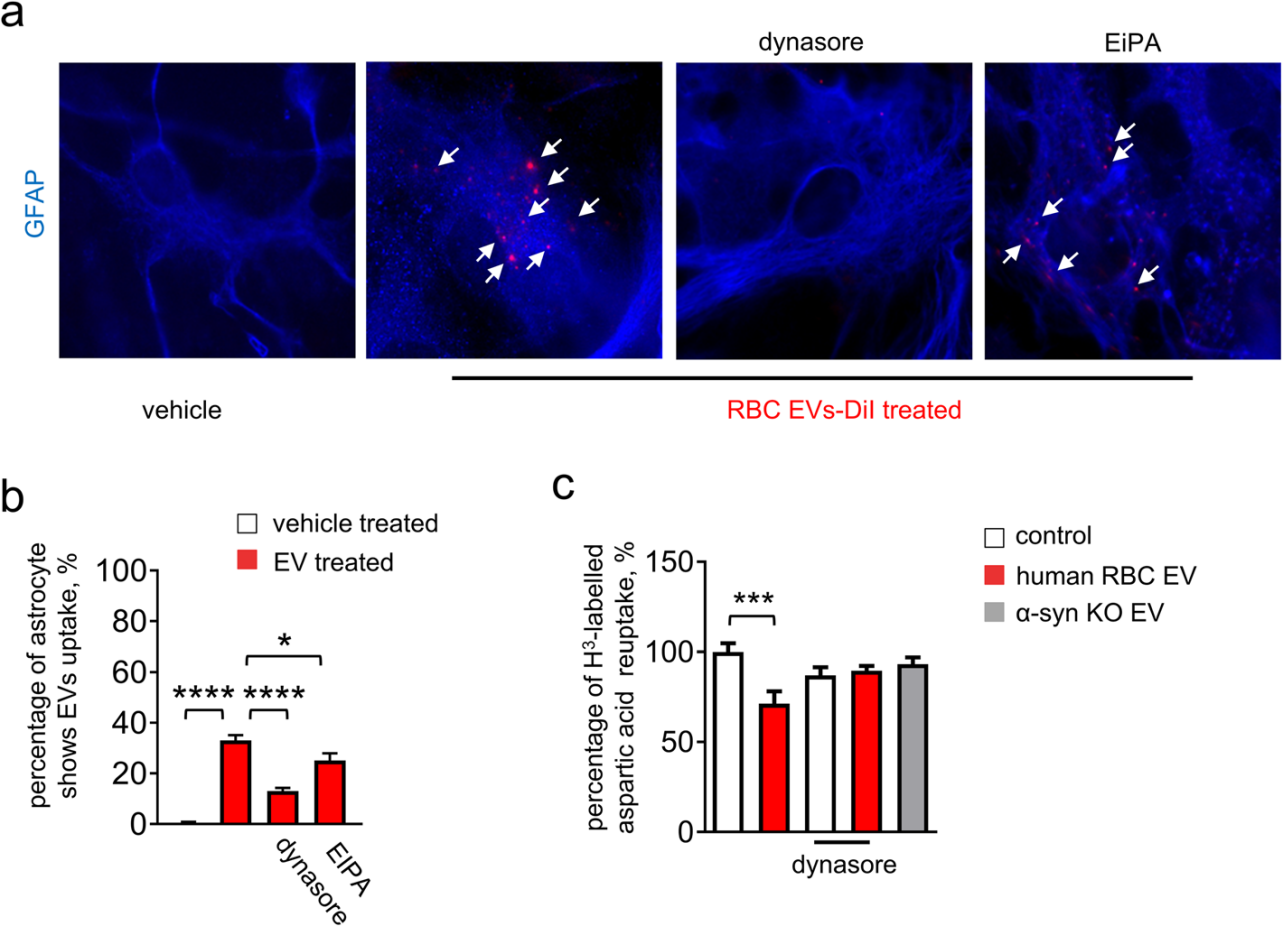
Astrocytes take up RBC-EVs via dynamin-dependent endocytosis resulting in dysfunction of glutamate clearance. **a** Representative images of cultured astrocytes treated with DiI-labeled RBC-EVs in the presence or in the absence of dynasore or EiPA. Application of dynasore prevented astrocyte uptake of RBC-EVs (Scale bar, 10 μm). **b** Quantification analysis of percentage of astrocytes showing RBC-EV uptake (means + S.E.M; *n* = 3; *p < 0.05, ****p < 0.0001 by One-way ANOVA followed by Tukey-Kramer’s post-hoc test). **c** Quantification analysis of uptake of H3-labelled aspartic acid in astrocytes. The presence of dynasore mitigated the effect of RBC-EVs (means + S.E.M; *n* = 3; ***p < 0.001 by ordinary Two-way ANOVA test and Sidak’s multiple comparisons post-test)

In order to further investigate the mechanisms by which RBC-EVs might affect astrocyte functions, we generated an in vitro model using cultured primary mouse astrocytes. Cultured astrocytes were confirmed to take up DiI-labelled RBC-EVs (Fig. 5a). To verify that the labeling observed in GFAP+ astrocytes corresponded to RBC-EVs, even after internalization, cultured astrocytes treated with DiI-labelled RBC-EVs were co-labeled with antibodies against human α-syn, which, in mouse astrocytes, may only be derived from the human RBC-EVs. DiI-RBC-EVs co-localized with human specific α-syn (Supplemental Figure 3a), suggesting that they were intact, as the lipid and protein content remained together. Next, to investigate the molecular pathway of astrocyte RBC-EV uptake, cultured astrocytes were pre-incubated with dynasore, an inhibitor blocking dynamin-dependent endocytosis, or EiPA, an inhibitor blocking micropinocytosis, before application of RBC-EVs. Analysis of immunofluorescence showed that EV uptake was blocked by dynasore, but not EiPA, suggesting that dynamin-dependent endocytosis, but not micropinocytosis, may be required **(**Fig. 5b).

Given the importance of glutamate homeostatic dysfunction in Parkinson’s disease [17, 34, 41, 47], we questioned whether glutamate clearance by astrocytes might be affected by toxic oligomeric forms of α-syn carried by RBC-EVs. We measured the effects of RBC-EVs on astrocytic glutamate clearance by performing an excitatory amino acid uptake assay in cultured primary astrocytes. ^3^H-labelled aspartic acid was added to the media of astrocytes treated with RBC-EVs in the presence or absence of dynasore. Treatment with RBC-EVs reduced the uptake of ^3^H-labelled aspartic acid in astrocytes by ~ 25% compared to control (Fig. 5c). The presence of dynasore mitigated this effect, indicating that internalization of EVs is necessary for the observed dysfunction to occur. Similarly, treatment with RBC-EVs derived from α-syn knock-out mice did not alter uptake of ^3^H-labelled aspartic acid when compared to untreated astrocytes (Fig. 5c), emphasizing that α-syn content is important for determining the detrimental effects of RBC-EVs on glutamate uptake in astrocytes.

**Fig. 6:**
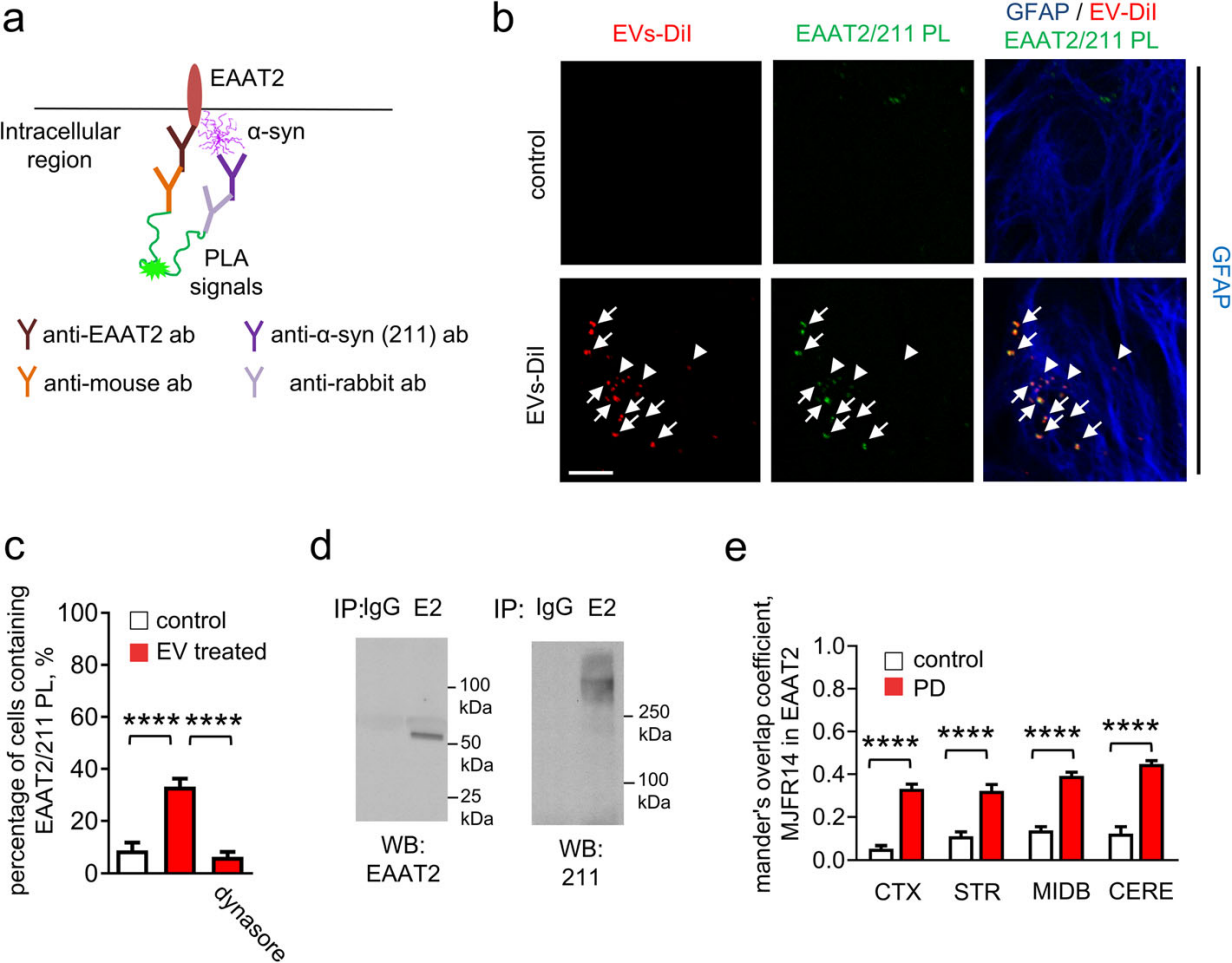
Interaction of α-syn and EAAT2 following RBC-EV exposure. **a** Schematic representation demonstrating the proximity ligation (PL) assay using antibodies against EAAT2 and human α-syn. **b** Representative images of cultured astrocytes containing EAAT2/α-syn complexes (proximity ligation products) treated with DiI-labeled RBC-EVs. White arrows indicate DiI-labeled RBC-EVs overlapping with proximity ligation products, white arrow heads indicate DiI-labeled RBC-EVs not overlapping with proximity ligation products (Scale bar, 10 μm). **c** Quantification analysis of percentage of astrocytes containing EAAT2/α-syn complexes (means + S.E.M; *n* = 3; ****p < 0.0001 by One-way ANOVA test). Note that the presence of dynasore eliminated the RBC-EV-induced increase of EAAT2/α-syn complexes. **d** Western blot analysis of EAAT2 (E2) immunoprecipitates (IP) from the lysates of A53T mouse brain performed with antibodies against EAAT2 and human α-syn (211). IP with control nonimmune rabbit immunoglobulins (IgG) served as control. **e** Quantification analysis of oligomeric α-syn/EAAT2 co-localization in cortex (CTX), striatum (STR), midbrain (MIDB) and cerebellum (CERE) (means + S.E.M; *n* = 5 independent animals were used in each group; ****p < 0.0001 by One-way ANOVA test)

We next sought to determine whether elevated α-syn might alter the function of EAAT2, which is responsible for removing over 90% of glutamate from synapses in CNS [36, 50]. To determine whether EAAT2 and α-syn might physically interact, we performed proximity ligation (PL) assay, in which a signal is only observed if the targets are in close physical proximity (Fig. 6a). The PL analysis was used in vitro with an antibody against the c-terminus of EAAT2, and Syn211, which specifically targets human-derived α-syn. We observed increased EAAT2/Syn211 (~ 40% of astrocytes) PL products in cultured astrocytes treated with RBC-EVs, when compared with untreated control or dynasore-treated astrocytes (Fig. 6c), indicating that these proteins were in close physical proximity, likely interacting. In contrast, no increase in PL signal was observed between α-syn and EAAT1, another glutamate transporter expressed in astrocytes (Supplemental Figure 3b). EAAT2/Syn211 products co-localized with DiI-labeled RBC-EVs along the processes of GFAP-labeled astrocytes (Fig. 6b). To confirm physical interaction between EAAT2 and α-syn, EAAT2 was immunoprecipitated from lysates of brain tissue from 4-month-old A53T mice. Western blot analysis of EAAT2 immunoprecipitates with antibodies against α-syn showed that oligomeric species of α-syn co-immunoprecipitated with EAAT2 (Fig. 6d), but not EAAT1 (Supplemental Figure 3c). We further sought to confirm this observation in vivo using A53T mice injected with either Parkinson’s disease or control RBC-EVs and found a significant increase in the oligomeric α-syn/EAAT2 co-localization (Fig. 6e).

**Fig. 7:**
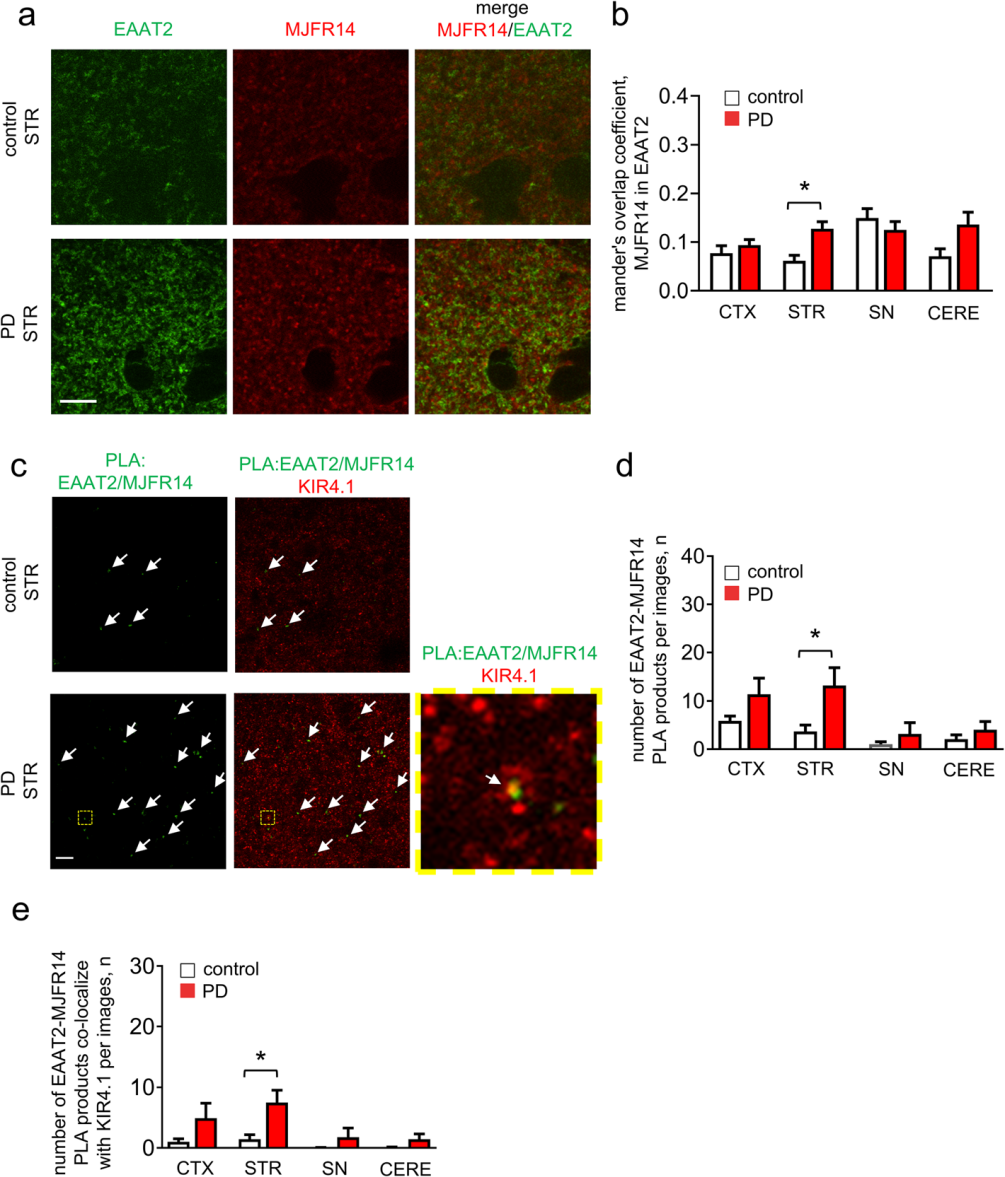
Oligomeric α-syn shows a close proximity with EAAT2 at the astrocytic endfeet in human brain tissue. **a** Representative images of human postmortem tissues (striatum (STR)) co-labeled for oligomeric α-syn and EAAT2. Note overlap of oligomeric α-syn with EAAT2 (Scale bar, 10 μm). **b** Quantification analysis of oligomeric α-syn/EAAT2 co-localization in cortex (CTX), striatum (STR), substantia nigra (SN) and cerebellum (CERE) (means + S.E.M; n ≥ 5; *p < 0.05 by One-way ANOVA test). **c** Representative images of human postmortem tissues (striatum (STR)) containing EAAT2/oligomeric α-syn complexes (proximity ligation products indicated by white arrows). Note that EAAT2/oligomeric α-syn complexes often co-localized with Kir4.1 labeled astrocytic endfeet (Scale bar, 10 μm). **d-e** Quantification analysis of number EAAT2/oligomeric α-syn complexes (**d**) or overlapping with Kir4.1 labeled astrocytic endfeet (**e**) in cortex (CTX), striatum (STR), substantia nigra (SN) and cerebellum (CERE) of healthy control or PD human postmortem tissues (means + S.E.M; *n* = 5; *p < 0.05 by One-way ANOVA test)

We further analyzed the distribution of oligomeric α-syn and EAAT2 in astrocytes in brain regions of Parkinson’s disease and control brains showing critical Parkinson’s disease pathological processes [11]. The immunofluorescence images showed that EAAT2 often co-localized with clusters of oligomeric α-syn in striatum in Parkinson’s disease brain tissues (Fig. 7a and Supplemental Figure 4a), suggesting that they are also in close proximity in humans, in agreement with our results in A53T mouse. We also performed the PL assay in our cohort of human brains (Fig. 7c-e). In Parkinson’s disease patients, the number of EAAT2/MJFR14 PL products was increased by ~ 2 folds in cortex and ~ 4 folds in striatum, when compared to healthy control subjects. Our results using human autopsy tissue demonstrate that α-syn interacts with EAAT2 at astrocytic endfeet, in alignment with our observations in the brains of A53T mice.

### RBC-EVs impair astrocytic protection of neurons

**Fig. 8:**
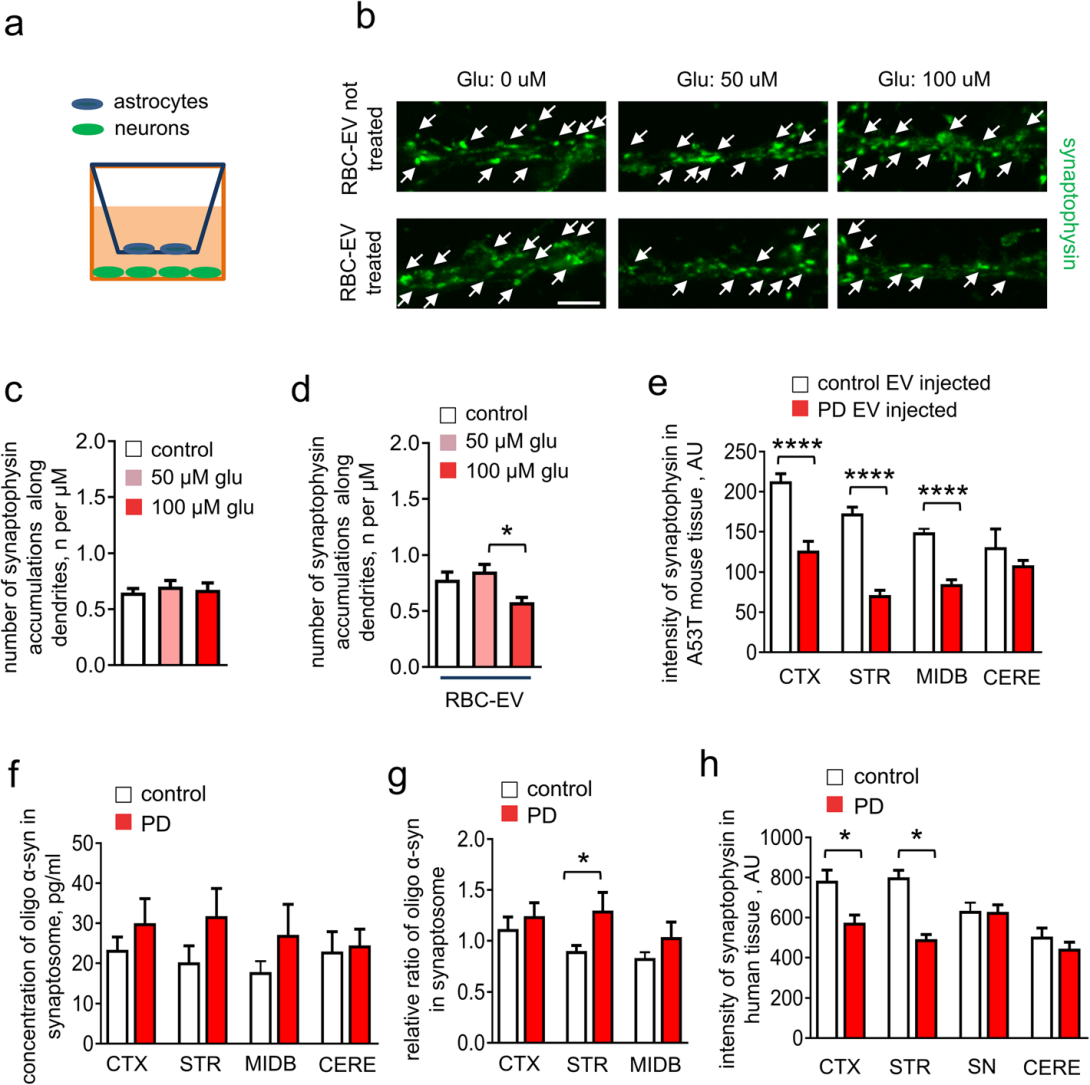
RBC-EVs impair astrocytic protection of neurons. **a** Schematic representation demonstrating the setup of the trans-well model. Astrocytes were cultured in the insert compartment of the trans-well and neurons were cultured in the bottom of the wells. **b** Representative images of cultured neurons labelled with synaptophysin. Note that RBC-EV pre-treated astrocytes cannot prevent the loss of synaptophysin-positive signals in the presence of glutamate treatment (Scale bar, 4 μm). **c-d** Quantification analysis of number synaptophysin puncta (means + S.E.M; *n* = 3; *p < 0.05 by One-way ANOVA). **e** Quantification analysis of fluorescence intensity of synaptophysin labeling in cortex (CTX), striatum (STR), midbrain (MIDB) and cerebellum (CERE) (means + S.E.M; *n* = 5; ****p < 0.0001 by One-way ANOVA test). **f-g** Graphs show the levels of oligomeric α-syn in synaptosomes of mouse cortex (CTX), striatum (STR), midbrain (MIDB) and cerebellum (CERE) after chronic RBC-EV injection (**f**) and normalized value against the concentration of oligomeric α-syn in cerebellum (**g**) (*n* = 5 independent animals were used in each group; means + S.E.M; *p < 0.05 by Student’s t-test *p < 0.05 compared to control-EVs injected group). **h** Quantification analysis of fluorescence intensity of synaptophysin labeling in cortex (CTX), striatum (STR), substantia nigra (SN) and cerebellum (CERE) of healthy control or PD human postmortem tissues (means + S.E.M; *n* = 5; *p < 0.05 by One-way ANOVA test)

Having found that astrocyte internalization of RBC-EVs led to oligomeric α-syn co-localization with EAAT2 and reduced aspartic acid uptake, we examined whether exposure of astrocytes to RBC-EVs impaired their ability to protect neurons from excitotoxicity using a trans-well culture model (Fig. 8a). Astrocytes cultured in the insert compartment were treated with either RBC-EVs or control media; after the excess (extracellular) RBC-EVs were washed out, the insert was transferred to wells in which primary neurons were plated. The co-culture arrangement was then treated with various concentrations of glutamate, none of which were toxic to neurons in the presence of astrocytes not exposed to RBC-EVs (no reduction of synaptophysin-positive signals, an index of neurodegeneration [13, 102], compared non-treated neurons; Fig. 8a-d) [5]. The count of synaptophysin-positive signals was reduced by ~ 30% in the presence of 100 μM glutamate in the RBC-EVs treated astrocytes, when compared with RBC-EV-treated astrocytes with 50 μM glutamate administration or without glutamate administration (Fig. 8b-d).

We also noted that fluorescence intensity of synaptophysin in striatum of mice injected with Parkinson’s disease RBC-EVs was reduced by ~ 50% when compared to control RBC-EV-injected mice (Fig. 8e). To corroborate these observations, synaptosomes were isolated from mouse brains after chronic EV injection [44]. MSD immunoassay measuring oligomeric α-syn detected increased oligomeric α-syn in striatum (~ 30%) from the Parkinson’s disease vs control RBC-EV-injected, while no significant effect was observed in cortex, midbrain or cerebellum (Fig. 8f and g), suggesting that astrocytic dysfunction mediated by RBC-EVs may further result in α-syn aggregation in or near synapses. In agreement with our observation, fluorescence analysis of Parkinson’s disease and control brains showed that synaptophysin was reduced in the striatum of Parkinson’s disease brain, compared to controls (Fig. 8h).

## Discussion

In this study, we made several novel observations, including: 1) compromise of the BBB in the A53T α-syn Parkinson’s disease mouse model permits entry of RBC-derived EVs in the absence of LPS; 2) oligomeric α-syn contained in RBC-EVs, particularly those isolated from Parkinson’s disease patients, may lead to oligomeric α-syn pathology in the endfeet of astrocytes (a phenomenon also observed in human autopsy tissue); and 3) RBC-EV influx likely leads to the disruption of astrocytic handling of glutamate, a critical step in metabolic homeostasis [86], which could culminate in neurodegeneration.

### Implications of peripheral α-syn contribution to Parkinson’s disease pathogenesis or progression

Mounting evidence unequivocally indicates that peripheral organs, e.g., the gastrointestinal system, heart, and skin, can be impaired at the earliest stages of Parkinson’s disease [19, 22, 51]. RBCs contain abundant α-syn [8, 81], and Parkinson’s disease patients exhibit morphological abnormalities of erythrocytes [69], possibly via the known effects of aggregated α-syn on cell membranes [38, 73]. Although its source is unknown, evidence published by other research groups [25, 64], and our previous study [94], showed that oligomeric α-synuclein can be even detected in plasma and red blood cells derived from healthy control subjects not demonstrating any Parkinson’s disease-related abnormalities in the CNS [64, 94, 99, 104]. In the present study, we show that RBC-derived EVs also contain oligomeric α-syn, which is increased in Parkinson’s disease patients, further suggesting the possibility that peripheral α-syn from RBC-EVs might represent an early driving source of pathological α-syn in Parkinson’s disease development and progression, as suggested previously [53].

In addition to peripheral pathology in Parkinson’s disease, it has been suggested that α-syn may also be transmitted from the periphery to the brain [35, 62, 63, 71, 72, 75, 98], which raises the possibility that peripheral components could contribute to the onset or progression of Parkinson’s disease. Most such studies to date, however, have been focused on the trans-synaptic spreading via nerves. Recent studies by us and others have indicated that systemic spreading of α-syn and other proteins directly from blood across the BBB may be an alternative pathway [6, 66, 89]. Our recent work demonstrated that RBC-derived, α-syn-carrying EVs could transfer from blood into the brain, likely via adsorptive-mediated transcytosis, particularly under LPS-induced systemic inflammation [53]. Our observation that A53T mice show BBB disruption at the early stage, in agreement with previous studies [30], further indicates that LPS-induced systemic inflammation is not required for RBC-EV transport in A53T mice even at early stages. To this end, there are multiple lines of evidence to suggest that the BBB is compromised in human diseases, including Parkinson’s disease [23, 29, 42, 91].

The BBB requires extensive support of multiple cell types, including astrocytes, to maintain its integrity [52], and abnormal astrogliosis mediated by toxic species of α-syn likely contributes to BBB disruption in Parkinson’s disease [15, 46]. When the BBB becomes dysfunctional, either via aging processes or secondary to a CNS pathology, peripheral α-syn can be important. In addition, a number of studies have identified an inflammatory response, accompanied by production of inflammatory cytokines, as a component of α-synuclein-induced astrogliosis [15, 46]. Astrocyte inflammatory activity modulates microglial behavior, in turn amplifying brain inflammation, and its toxic effects on neurons, as well as having consequences for BBB permeability [3, 21].

### Oligomeric α-syn from the blood is taken up by astrocytes

Multiple studies have demonstrated that astrocytes take up oligomeric α-syn likely derived from the CNS [15, 32, 74], leading to neuroinflammation and neuronal toxicity. Uptake of peripheral oligomeric α-syn by astrocytes is a novel pathway revealed in this study. In our previous investigation [53], we observed that RBC-EVs could be transported into the brain after LPS-treatment, and are largely colocalized with Iba-1+ microglia, not with GFAP+ astrocytes or MAP 2+ neurons. In contrast, in the present study, periphery-derived oligomeric α-syn-containing RBC-EVs were taken up by astrocytes, mostly at the endfeet surrounding the BBB. A few major differences between our studies may help account for this obvious discrepancy. First, the uptake of EVs by astrocytes was assessed using co-localization of EVs with GFAP in our previous investigation. However, GFAP is expressed primarily in the somata and proximal processes of astrocytes, limiting its utility to measure the co-localization of EVs with the distal processes, the part of the cell in closest contact with the BBB, and most likely site of initial EV internalization [86]. Indeed, astrocytic endfeet cover the major area of vascular surfaces [58, 68, 84] in addition to shaping the BBB. These interactions play a critical role in regulating the exchange between peripheral blood flow and CNS activities [4]. In the current study, using a marker (Kir4.1) more sensitive for astrocyte processes, we revealed evidence for astrocyte uptake of RBC-EVs. This result also explained a phenomenon in our previous study, i.e. some signals could not be associated with a particular cellular marker [53]. As to the difference in neuronal uptake, it should be noted that, in both studies, neuronal uptake makes up a very small proportion of EVs (3–11%, depending on region) and thus a precise comparison in neuronal uptake between the two experiments is quite challenging. To further complicate the issue, in our previous study, LPS was used to induce systemic inflammation to facilitate the entry of EVs into the brain. It is well-known that LPS results in potent microglial activation [37], which could promote microglial uptake of RBC-EVs while suppressing the uptake by other cell types. Indeed, in the current study, we observed a larger proportion of EVs in the striatum and midbrain of LPS-treated animals compared with A53T animals, further supporting the possible hypothesis that LPS and α-syn-related stress might result in different responses of brain cells to RBC-EVs. Finally, in our previous study, 8-week old (~ 2 months old) mice were used for RBC-EVs injection, while in this study, 3-month old mice were utilized because it takes time for A53T mice to develop CNS pathology. To this end, aging has been proposed as a key modulator in altering physiological function of the BBB and brain cells [91, 92]. In other words, it is quite possible that the distribution of RBC-EVs may heavily depend on the age of mice in a given study.

As described above, by way of physical proximity, astrocytes are among the first cells in the brain to contact periphery-derived α-syn crossing the BBB. This, along with their established ability to take up α-syn in vitro [46, 83], may explain the robust entry of RBC-EVs and associated oligomeric α-syn into astrocyte processes. Interestingly, although RBC-EVs could be taken up by astrocytes in different brain regions (cortex, striatum, and midbrain), our results show that the preferential uptake of RBC-EVs in astrocytic endfeet is particularly pronounced in striatum (Fig. 3), an early site of Parkinson’s disease pathogenesis [11, 12], in A53T animals, in agreement with previous evidence demonstrating significantly increased permeability of the BBB in striatum of Parkinson’s disease patients [29]. Furthermore, following a sub-chronic exposure to exogenously applied RBC-EVs in the present study, significant changes in oligomeric α-syn levels (Fig. 2c-f) were more apparent in striatum, either in the tissue as a whole (immunoassays shown in Fig. 2) or in specific cells (Fig. 4), suggesting that the accumulation of α-syn aggregates might be correlated with the amount of RBC-EV uptake by astrocytes. Also, it has been reported that early stages of Parkinson’s disease feature degeneration of nigrostriatal dopaminergic terminals in the striatum, prior to the loss of the cell bodies in the substantia nigra [22, 26, 27, 39, 96]. Thus, our observations of the preferential uptake in astrocytic endfeet and accumulation of toxic α-syn species in striatum is in alignment with the concept of the involvement of the nigrostriatal pathway in Parkinson’s disease development. Notably, when the effect of EV uptake on oligomeric α-syn expression was measured with greater spatial resolution (that is, by cell type, comparing cell body to processes in astrocytes) in Fig. 4, multiple regions showed significant differences in oligomeric α-syn, supporting the notion that α-syn pathology may further spread into other brain regions following infiltration of oligomeric α-syn-containing EVs, which might represent a process similar to that resulting in the progression of Parkinson’s disease in humans.

Another major discovery of this study is that chronic injection of Parkinson’s disease RBC-EVs in mice resulted in accumulation of oligomeric α-syn, the toxic species of α-syn, prominently in the astrocytic endfeet, suggesting that this may be the major, or initial, locus of pathology transferred to the brain by RBC-EVs during BBB disruption. This finding is consistent with the observation of α-syn aggregations localized at the astrocytic endfeet in postmortem Parkinson’s disease brains [12, 100]. However, the relative abundance of astrocyte α-syn arising from different sources (i.e., neuronal, peripheral, or endogenously expressed) remains to be determined. Regardless of what proportion of astrocytic α-syn is derived from peripheral sources, in agreement with our observation, a recent study demonstrated evidence that peripheral injection of blood EVs derived from aged mice activate astrocytes [55], further indicating that astrocytes play a critical role in response to peripheral-derived EVs. In addition, stroke-induced enhanced BBB permeability promotes EVs and pro-inflammatory factors passing through the BBB, resulting in abnormal activation of astrocytes [59, 103]. This evidence further suggests that astrocytes play a role as a defense system in response to BBB disruption and peripheral insults, and their responses to insult may in turn exacerbate the condition of the brain.

Taken together, astrocytes play a potential role in taking up peripheral RBC-EVs, which may act as the starting point of the molecular mechanisms contributing to the progression of α-syn pathology in Parkinson’s disease.

### Oligomeric α-syn disrupts glutamate handling leading to the loss of synapses

One of the critical functions of astrocytes is to rapidly remove excess glutamate in the extracellular space to prevent neuronal excitotoxicity [49], a process that has been highlighted in Parkinson’s disease [97, 105]. Here, we observe a novel mechanism by demonstrating a direct interaction between oligomeric α-syn and EAAT2, one of the key machineries in recycling glutamate. Our in vivo results show that long-term injection of RBC-EVs derived from Parkinson’s disease patients resulted in increased interaction between oligomeric α-syn and EAAT2 at the astrocytic endfeet. Consistently, increased association between oligomeric α-syn and EAAT2 were observed at astrocytic endfeet in Parkinson’s disease post-mortem brain tissues, suggesting that dysfunction of glutamate clearance in astrocytes is likely a detrimental result of oligomeric α-syn spreading from peripheral circulation.

While the result obtained in autopsy tissue is correlational, in a previous observation, along with BBB leakage and the development of α-syn aggregation in the endfeet of astrocytes at the early stage of development, A53T mice demonstrated direct evidence of dysfunction of glutamate uptake in astrocytes [30], further supporting the argument that RBC derived-α-syn provokes dysfunction of the glutamate homeostasis. Relatedly, it is well-known that an essential event in neurodegeneration, including Parkinson’s disease, is represented by loss of synapses on neurites [2, 18]. Previous studies have demonstrated that synaptophysin levels are unchanged in A53T mice compared to control mice [85]. In addition, in vitro evidence suggested that overexpression of A53T α-syn in culture neurons did not affect the density of synaptophysin accumulations [93]. These suggest that A53T α-syn overexpression alone might not be able to induce similar changes in RBC-EVs (and thus further changes in synapses) to those in Parkinson’s disease-derived RBC-EVs. Indeed, introduction of RBC-EVs from human Parkinson’s disease patients resulted in loss of synapses compared to those from control subjects, along with accumulations of oligomeric α-syn. Given that the synaptic dysfunction in Parkinson’s disease is likely associated with vulnerability of human dopaminergic terminals, further resulting in abnormal dopamine metabolism and depletion in dopamine neurotransmission, this finding is in alignment with the human condition, particularly given our observation in human tissue of loss of synaptophysin in striatum. The observed astrocytic dysfunction of glutamate re-uptake mediated by RBC-EV uptake is likely to facilitate the loss of dopaminergic terminals, as evidenced by the loss of synapses, along with neurites in a region-specific manner. Further studies are needed to confirm whether dopaminergic terminals are particularly vulnerable to astrocytic uptake of RBC-EVs. One caveat, however, is that it is difficult to assess the effects of RBC-EVs on healthy brains, as entry of RBC-EVs into the brains of WT animals with intact BBB (i.e., not disrupted by LPS injection or other treatments) is exceedingly low, and alternative methods for introducing them into the brain (e.g., injection) would not preserve the anatomical distribution that may contribute to astrocyte uptake in the distal processes.

## Conclusion

Our observations show that BBB disruption during early stages of Parkinson’s disease pathogenesis allows the transport of oligomeric α-syn containing RBC-EVs from peripheral circulation into the CNS, leading to or enhancing oligomeric α-syn pathology in the endfeet of astrocytes. The resulting abnormality of astrocytic function likely involves disruption of astrocytic handling of glutamate via the interaction between excess α-syn and EAAT2. Although the cross-talk between peripheral α-synucleinopathies and astrocyte dysfunction-dependent α-syn spreading still requires further investigation, this potential new pathway further emphasizes that initiation of α-synucleinopathies is likely derived from a set of complex mechanisms and the function of astrocytic endfeet in their role as a component of the BBB may be a key to understanding the initiation and progression of Parkinson’s disease.

## Materials and methods

### Animals

A53T mice (B6;C3-Tg (Prnp-SNCA*A53T)83Vle/J) [28], α-syn knock out mice (B6;129X1-Snca<tm1Rosl>/J) [80], and their wild-type controls of either sex were purchased from Jackson laboratory for RBC-EV injection studies. The genotype of all A53T mice was confirmed by using genomic DNA samples isolated from mouse tails and RT-PCR following the animal provider’s instructions. Additionally, brain tissues from 1- to 3-day-old C57BL/6 mice of either sex were used to prepare cultures of primary astrocytes and primary cortical neurons. All animals were kept and bred on a 12/12-h light/dark cycle with ad libitum food and water. Experiments were approved by the Institutional Animal Care and Use Committee of the University of Washington (IACUC number: PROTO201600894: 3439–01).

### Human subjects and clinical sample collection

All participants underwent detailed informed consent procedures and provided consent in writing in accordance with procedures approved by the institutional review boards at the Veterans Affairs Puget Sound Health Care System and the University of Washington (IRB: STUDY00003047).

Plasma samples were collected from 109 patients with Parkinson’s disease and 59 age- and sex-matched healthy controls as previously described [80]. All Parkinson’s disease patients and 9 of the controls were enrolled in the Pacific Udall Center Clinical Core which includes sites at the University of Washington/Veterans Affairs Puget Sound Health Care System, and Oregon Health Sciences University/Veterans Affairs Portland Health Care System. The remainder of the controls were from the University of Washington Alzheimer’s Disease Research Center neuropathology core. All subjects underwent evaluations consisting of medical history, physical and neurological examinations, laboratory tests, and neuropsychological assessments. All Parkinson’s disease patients were determined to meet UK Parkinson’s Disease Society Brain Bank clinical diagnostic criteria for Parkinson’s disease as determined by a consensus of movement disorder specialists [16]. Control subjects were in good health without evidence of a neurodegenerative disorder. Similar sample collection protocols and quality control procedures were followed at all participating sites as previously described [80], including key steps such as use of polypropylene collection and storage tubes, rapid separation into single use aliquots, and freezing of plasma samples, to minimize potential site variability. Demographic information of all subjects is listed in Supplemental Table 1, and the plasma pooling strategy for RBC-derived EV injection experiments is listed in Supplemental Table 2.

Human reference plasma was generated by pooling ~ 30 healthy controls for optimization of the RBC-EV immuno-capture method. Human erythrocytes from healthy control subjects were also purchased from ZenBio (Research Triangle Park, North Carolina) for isolation of EVs from cultured erythrocytes.

Post-mortem human brain specimens were provided by the University of Washington Alzheimer’s Disease Research Center neuropathology core. Formalin-fixed paraffin embedded human brain specimens of 5 Parkinson’s disease patients and 5 age−/sex-matched control subjects were used. Specimens containing the frontal cortex, the corpus striatum, substantia nigra, and cerebellum were cut to a thickness of 7 μm and tissue sections were mounted onto glass microscope slides. The demographic information is listed in Supplemental Table 3.

### EV isolation

The method for human RBC-derived EV isolation from cultured human erythrocytes was previously described [53]. Briefly, human erythrocytes from healthy controls were cultured in RPMI-1640 culture medium containing 25 mM HEPES at 37 °C with 5% CO_2_ for 48 h. Culture medium was collected and centrifuged at 1500×g for 10 min, followed by centrifugation at 3000×g for 15 min (2 times) to remove the intact cells, cell debris and apoptotic blebs. Supernatants were collected and subjected to centrifugation at 150,000×g for 2 h. The resulting pellet was then washed with ice cold PBS, followed by centrifugation at 200,000×g for 2 h. The EV pellet was resuspended and collected using ice cold PBS. Next, the EV sample was purified using a sepharose CL-2B column (Sigma, USA). 0.5 mL fractions were collected in 1.5 mL tubes and the protein concentration of each fraction was measured based on UV absorbance at 280 nm using a NanoDrop™ Lite Spectrophotometer (Thermo Fisher Scientific, USA). Two observed protein peaks (Peak 1 and Peak 2; EVs were enriched in Peak 1 as described previously [53]) were further concentrated using Amicon® Ultra centrifugal filter devices (cut-off MW 100 kDa, Millipore Corporation, USA).

To allow the use of archived plasma samples from patients with Parkinson’s disease and healthy controls, RBC-EVs were also isolated from human plasma using an immuno-capture method developed by our laboratory [80]. Briefly, 10 μg of antibodies against CD235a or IgG isotype controls were coupled onto one set (1 mg) of M-270 Epoxy beads in alignment with the manufacturer’s instructions (Dynabeads® Antibody Coupling Kit, Thermo Fisher Scientific, USA). Plasma samples were thawed at 37^o^ C, followed by centrifugation at 2000×g for 15 min and 12,000×g for 30 min. Supernatant of plasma was diluted in PBS (pH 7.4) at a 1:3 ratio. Next, one set of antibody-coated beads and 900 μL of diluted plasma were incubated for ~ 24 h at 4 °C with gentle rotation. After the incubation, beads were washed four times using 1 mL of 0.1% BSA/PBS (pH 7.4) to remove non-specific binding and transferred into a new tube before elution. For immunoassays, EVs were eluted from the beads and lysed by incubating the beads in 110 μL of 1% Triton X-100 plus 10% of a protease inhibitor cocktail (P2714, Sigma-Aldrich;prepared in 10 ml of H2O) in 0.1% BSA/PBS (pH 7.4). For NTA, animal injection, and EM, EVs were eluted from the beads using 70 μl 0.1 M glycine followed by a room temperature incubation on a vortex shaker for 15 min. The supernatant was neutralized by adding 5 ul of 1 M Tris (pH 7).

### RBC-EV injection in mouse

The RBC-EVs isolated from cultured human RBCs or from human plasma were labeled with Vybrant™ DiI cell-labeling solution in accordance with the manufacturer’s instructions (Thermo Fisher, USA), which were previously described [53]. Briefly, RBC-EVs were re-suspended in 500 μL of PBS followed by the application of DiI solution (10^− 3^ μmol). Then, RBC-EVs were incubated with DiI solution for 20 min at room temperature. Next, excess DiI dye was removed and the labeled EVs were concentrated using Amicon® Ultra centrifugal filter devices (cut-off MW 100 kDa). Finally, the EVs were washed three times using PBS, and resuspended in 50 μL of PBS before use.

The distribution of intravenously (i.v.) injected RBC-EVs in mice in vivo was assessed in live animals and by immunofluorescence. Some animals underwent an LPS pre-injection procedure previously demonstrated to allow RBC-EVs to cross from the blood into the brain in WT mice [53]. Three month old animals of either sex were weighed and given an intraperitoneal injection of LPS (3 mg/kg dissolved in sterile normal saline) derived from Salmonella typhimurium (Sigma Aldrich) or control saline three times (at 0, 6 and 24 h), followed by a 28 h waiting period before RBC-EV injection. Vybrant™ DiR cell-labeling solution were added to the RBC-EVs according to the manufacturer’s instructions, and DiR-labeled RBC-EVs dissolved in PBS (50 μg per mice) or control (PBS without EVs) were intravenously injected via the tail vein to LPS (3 mg/kg of mice, *n* = 5) or control saline pre-injected (i.p. injection) mice (*n* = 5). The heads of the mice were shaved to reduce potential interference, prior to injection of RBC-EVs. Mice were anesthetized with 2% isoflurane during imaging with an IVIS Spectrum in vivo imaging system (PerkinElmer, USA).

For immunofluorescent analysis of RBC-EV distribution, animals were pre-treated with LPS and injected with RBC-EVs as above, then perfused after 3 h. The descending aorta was clamped and both jugular veins were severed. An 18-gauge butterfly needle was injected into the left ventricle of the heart and then PBS infused at a rate of 2 ml/min for 5 min, followed by perfusion of 4% paraformaldehyde solution at a rate of 2 ml/min for 5 min. Brains were removed and immersed in 4% paraformaldehyde solution at 4 °C overnight. After dehydration in 20% sucrose, sagittal brain sections (20 μm) were prepared with a sliding microtome (Leica, Wetzlar, Germany).

To investigate chronic exposure to RBC-EVs, RBC-EVs obtained from the plasma of human Parkinson’s disease patients or healthy controls were labeled with DiI, and injected 2 times/week i.v. [50 μg EVs (proteins) per mice] into 3 month old A53T mice (*n* = 5 for each group) for 6 weeks via tail vein. Animal weight was recorded every week without showing significant weight loss during the injection. Then, mice were perfused, and brain tissues were collected as described above.

### Nanoparticle tracking analysis (NTA)

The number of particles and size distribution in RBC-EV preparations were analyzed with NTA (NS300; Nanosight, Amesbury, UK). For RBC-EVs derived from cultured fresh RBCs, each fraction was diluted 1000-fold in PBS in order to optimize the number of particles counted. For EVs isolated from plasma using the immuno-capture method, eluted EVs were diluted 10-fold in PBS, followed by NTA analysis. For each fraction, three videos (60 s each) were captured, and all fractions were analyzed using the same threshold. Analysis was performed by NTA 3.2 software (Nanosight, Amesbury, UK).

### Primary neuron and astrocyte cultures

The cultures were established as described previously [77, 101] with modifications. Briefly, primary cortical astrocytes were prepared from postnatal day 0 to 3 mice. Cortices were isolated and cleaned of blood vessels and meninges in cold dissecting media (Dulbecco’s modified Eagle’s medium F12, Thermo Fisher, USA). Tissue was digested for 30 min at 37 °C in Dulbecco’s modified Eagle’s medium F12 with 0.5 mmol/L EDTA, 0.2 mg/mL l-cysteine, 15 U/mL papain (Worthington Biochemical, USA), and 10 μg/mL DNase (Worthington Biochemical, USA) (10-mL digestion media per three brains). After digestion, cortical tissue was washed three times with culture media [Dulbecco’s modified Eagle’s medium F12 supplemented with 10% fetal bovine serum and 1% penicillin/streptomycin (Corning, Tewksbury, USA)]. After washing, cortices were triturated with a fire-polished Pasteur pipette in 10 mL of culture media. Tissue was allowed to settle, and the supernatant was passed through a cell strainer (pore size, 100 μm). The remaining tissue was triturated again in 5 mL of culture media, passed through a strainer, and combined with the previous 10 mL of triturated cells. The resulting cells were seeded in culture media, into vented 75-cm^2^ flasks coated with poly-d-lysine, two brains per flask. Culture medium was changed 24 h later, and astrocytes were maintained at 37 °C, 5% CO2, until they reached confluence (9 to 10 days), at which point they were plated for use.

Flasks containing confluent astrocyte cultures were gently shaken to remove microglia, washed once with PBS, and incubated with 0.25% trypsin EDTA at 37 °C for approximately 5 min. Cells were washed with an equal volume of media, collected into a 50-mL conical tube, and centrifuged at 1600×g for 10 min at room temperature. The resulting pellet was resuspended to a final volume of 10 mL per flask collected and counted using a hemocytometer. Cells were plated onto insert wells (for co-culture, 1 × 10^5^ cells per coverslip) or into 24-well plates (1 × 10^5^ cells per well). Cultures were at least 99% glial fibrillary acidic protein (GFAP) positive.

For neuronal cultures, cortices of mouse brains from postnatal day 0 to 3 mice were placed in cold neuronal dissection media [Neurobasal A supplemented with B27 and 0.5 mmol/L l-glutamine (Thermo Fisher, USA)]. Ventral mesencephalons were dissected from the brains and digested for 15 min at 30 °C in 12 mL Hibernate A without calcium chloride (BrainBits, USA), 0.5 mmol/L l-glutamine, 15 U/mL papain, and 17 μg/mL DNase. Tissue was washed three times with neuronal dissection media and triturated in 2 mL of the same media with a fire-polished Pasteur pipette. Tissue was allowed to settle, and the supernatant was passed through a cell strainer (pore size, 100 μm). Trituration and cell straining were repeated twice more. Cells were spun at 1000×g for 5 min at room temperature. The resulting pellet was resuspended in neuronal media (Neurobasal A supplemented with B27, 2 mmol/L l-glutamine, and 1% penicillin/streptomycin) and counted using a hemocytometer. Cells (1.5 × 10^5^ per well) were plated into 24-well plates. Neurons were allowed to grow for 14 days before experiment. Changes of culture media were performed on days 2, 3, 6 and 12.

### Immunofluorescence

Mouse brain slice staining was performed as described previously [53]. Mouse brain slices were washed with PBS and treated with blocking solution (1% BSA, 0.4% Triton X-100 and 4% goat serum in PBS) for 1 h at room temperature followed by incubation overnight at 4 °C with primary antibodies (anti-GFAP (PA1–10004, Thermo Fisher Scientific, USA); anti-Kir4.1 (KCNJ10, Alomone labs, USA); anti-EAAT2 (MAB 2262; Millipore Sigma, USA); α-synuclein filament (MJFR14–6–4-2, Abcam, USA) anti-synaptophysin (D-4, sc-17,750, Santa Cruz Biotechnology, USA)) diluted in blocking solution. Brain slices were washed using washing buffer (0.1% Tween in PBS) and incubated with corresponding secondary antibodies diluted in PBS containing 0.3% of Triton X-100 for 2 h. After washing with PBS or washing buffer, brain slides were embedded in Vectashield medium or Vectashield medium with DAPI (when indicated).

Human postmortem tissue staining was performed as described previously [33]. Fixed and paraffin-embedded tissue sections were deparaffinized by washing 4 × 10 min in xylene and 2 × 5 min in 50% xylene/50% ethanol. Tissue was rehydrated by washing 2 × 5 min in 100% ethanol, 3 × 1 min in 95, 70, and 50% ethanol, rinsed in deionized water, and washed 2 × 5 min in phosphate-buffered saline (PBS). Tissue was heated (≥100 °C) in 10 mmol/L citric acid (pH 6.0) for 15 min, cooled for 30 min at room temperature (25 °C), and washed 3 × 10 min with TBS-T. Tissue was blocked overnight in 5% normal goat serum (NGS), 2% BSA, and 0.25% Triton X-100, made in TBS-T. Tissue was incubated with primary antibodies (anti-GFAP (PA1–10004, Thermo Fisher Scientific, USA); anti-Kir4.1 (KCNJ10, Alomone labs, USA); anti-EAAT2 (MAB 2262; Millipore Sigma, USA); α-synuclein filament (MJFR14–6–4-2, Abcam, USA); anti-synaptophysin (D-4, sc-17,750, Santa Cruz Biotechnology, USA); chicken antibodies against MAP 2 (ab5392, Abcam, USA)) in blocking solution overnight. After incubation with primary antibodies, tissue was washed with 5% NGS and 2% BSA in TBS-T 3 × 10 min, and incubated with secondary antibodies conjugated with Alexa Fluor 488 or 568 (dilution 1:500; Life Technologies, Carlsbad, CA) in 5% NGS and 2% BSA, made in TBS-T, overnight. After incubation with secondary antibodies, tissue was washed 3 × 10 min in TBS-T, followed by rocking in 0.3% Sudan Black in 70% ethanol for 30 min. Tissue was rinsed twice in 70% ethanol, washed 3 × 10 min in TBS-T, and mounted with Vectashield with DAPI (Vector Laboratories, USA).

Immunocytochemistry was performed as described previously [78]. Cells were fixed in 4% paraformaldehyde for 30 min at room temperature, washed 2 × 5 min with PBS, and blocked in 4% NGS, 1% BSA, and 0.4% Triton X-100 for 1 h at room temperature. Primary antibodies (anti-GFAP (PA1–10004, Thermo Fisher, USA; anti-synaptophysin (D-4, sc-17,750, Santa Cruz Biotechnology, USA); anti-α-syn (clone 42, 624,096, BD Bioscience, USA)) were diluted in blocking buffer and incubated overnight at 4 °C. Astrocytes fixed after mitochondrial imaging were stained with primary antibodies following with overnight incubation at 4 °C. Cells were washed 3 × 5 min with 0.1% Tween-20 in PBS and incubated with secondary antibodies conjugated with Alexa Fluor 488, 568, or 633 diluted 1:500 (488 and 568) or 1:100 (633) (Thermo Fisher, Carlsbad, CA; Abcam, USA) in 0.3% Triton X-100 in PBS for 1 h at room temperature, followed by washing 3 × 5 min with 0.1% Tween-20 in PBS. Stained cells were mounted with Vectashield with DAPI. Immunofluorescence images were captured by imaging with an oil-immersion Plan Apo VC × 60 objective using a laser-scanning confocal microscope (Olympus, USA). Z-series images were acquired from randomly selected fields in the presence or in the absence of DiI-labeled RBC-EVs. Note that DiI signal in the brain may signify that EV membrane is present, but cannot inform on the number of EVs or their cargo.

Captured images were analyzed using Fiji software, which is a derivative of ImageJ [76]. For the quantitative analysis of fluorescence intensity, three square fields (size 20 μm^2^) including GFAP-positive signal, were randomly selected (excluding any artifacts) for each image or region of interest (ROI), followed by measurement of fluorescence intensity of the selected area. Then, fluorescence intensity of the background, selected manually in each image outside the positive area, was deducted from the intensity of the signal. For the co-localization analysis, square ROI (size 20 μm^2^) were randomly selected for the two channels of interest, followed by calculation of Mander’s overlap coefficient between the analytes in the selected ROI, which has been described previously [24]. The DiI-signals, representing the labeling of RBC-EVs, were defined as a mean intensity of at least 10% higher than background labeling, which were manually counted and analyzed as described previously by our laboratory [53].

### Proximity ligation (PL) assay

PL assays in cultured cells were performed as described previously [48, 78] with modifications. Cultured astrocytes were treated with RBC-EVs at 37 °C for 3 h. Then, cultured astrocytes were fixed in 4% formaldehyde in PBS, washed with PBS and blocked by incubating with blocking solution (5% donkey serum, 2% BSA and 0.1 Triton x-100 in PBS) for 1 h. Primary antibodies against the intracellular domain of EAAT2 and α-syn were applied to the cells in 0.1% BSA in blocking solution overnight at 4 °C. Further steps were performed using secondary antibodies conjugated with oligonucleotides (PLA probes anti-mouse minus and anti-rabbit plus, DUO92004 and DUO92002, Sigma Aldrich, USA) and Duolink II fluorescence kit (DUO92014, Sigma Aldrich, USA) in accordance with the manufacturer’s instructions. Fluorescence images were acquired at room temperature using a confocal laser scanning microscope (Olympus, PA, USA) and oil Plan Apo VC × 60 objective (numerical aperture 1.4). For human postmortem brain tissues, after overnight incubation with primary antibodies, modification of manufacturer’s instructions was applied at the ligation and amplification steps: time of incubation with the ligase buffer was increased to 90 min and time in the polymerase buffer was increased to 2 h. Fluorescence intensities of PL products in cultured astrocytes and human postmortem brain tissues were measured in Fiji and verified by 3 independent investigators.

### Electrochemiluminescence (ECL) immunoassays for total and aggregated α-syn quantification

The ECL immunoassays were performed as previously described and validated [94] with minor modifications for human RBC-EV and mouse brain tissue homogenate samples in this study. Antibodies against α-syn clone 42 (624,096, BD Bioscience, San Jose, CA, USA) were labelled with sulfo-TAG reagent in alignment with MSD instructions, and used as the detector for both assays. Antibodies against α-syn MJFR-1 clone 12.1 (ab138501, Abcam, USA) and antibodies against conformation specific, α-syn filaments MJFR-14 (ab209538, Abcam, USA) were independently biotinylated, linker conjugated, and coated onto standard 96-well U-Plex plates (MSD, USA) by incubating the plates with 10 μg/ml of capture antibody solutions for 2 h at room temperature with 600 rpm shaking. After washing three times with 150 μl Washing Buffer (MSD, USA), plates were blocked with 150 μL Diluent 35 (MSD, USA) for 1 h while shaking at 600 rpm at room temperature, following with three washes using Washing Buffer. Immuno-captured RBC-EV samples were subjected to a 1:2 dilution using Diluent 35, and brain tissue homogenates were diluted 1:20 with Diluent 35. Then, 50 μl of diluted sample and calibrator (recombinant α-syn, Proteos, USA, and oligomeric α-syn [94] were loaded to the immunoassay plate following with 1 h room temperature incubation on a shaker at 600 rpm. Next, the plate was washed three times using Washing Buffer and sulfo-TAG-labelled anti-α-syn clone 42 antibody (1μg/ml) was added along with room temperature incubation for 1 h on a shaker at 600 rpm. Finally, the plate was washed three times using Washing Buffer and 150 μL of Read Buffer T (MSD, USA) was applied to each well, and plates were analyzed in a Quickplex SQ 120 (MSD, USA). Data analysis was performed with the MSD Discovery Workbench 3.0 Data Analysis Toolbox.

### ^3^H-d-aspartate uptake assay

Uptake assays were performed on cultured astrocytes, as described previously [33]. Astrocytes were first incubated in the presence or in the absence of dynasore for 3 h at 37 °C, followed by application of RBC-EVs and incubation for 3 h at 37 °C. Then, astrocytes were washed twice with warm Krebs-Ringer solution (16 mmol/L sodium phosphate, 119 mmol/L NaCl, 4.7 mmol/L KCl, 1.8 mmol/L CaCl2, 1.2 mmol/L MgSO4, 1.3 mmol/L EDTA, and 5.6 mmol/L glucose; pH 7.4). After the second wash, cells were incubated with Krebs-Ringer solution with ^3^H-d-aspartate (1.3 μmol/L, 0.03 μCi/mL, specific activity 1 mCi/mL; American Radiolabeled Chemicals, St. Louis, MO) at 37 °C for 10 min. Astrocytes were washed three times with ice-cold Krebs-Ringer solution. After the third wash, astrocytes were rocked in 1 N NaOH for 30 min at room temperature. The resulting solutions were collected in scintillation fluid and measured using a scintillation counter. For each replicate, ^3^H measurements were averaged for each group and normalized to the value for astrocytes without treatment (control group).

### Electron microscopy (EM)

RBC-EV sample preparation was conducted based on a previously described protocol [65]. Briefly, RBC-EV samples were pre-incubated with antibodies against CD63 (556,019, BD Biosciences, USA) overnight at 4 °C, followed by incubation with 10-nm gold conjugated goat anti-mouse IgG antibody (abcam) for 60 min at room temperature. Then, samples were applied to glow-discharged holey grids, following with loading and blotting of 10 μl RBC-EV samples for 5 min (repeated 3 times). Grids with EV samples were plunged in liquid ethane using a Vitrobot (FEI) and maintained in liquid nitrogen until imaging. Images were acquired and collected on a FEI Tecnai G2 F20 (FEI Co, USA) at 200 kV Cryo-S/TEM equipped with a Gatan K-2 Summit Direct Detect camera. Leginon software was used to automate data collection [90].

### Western blot and dot-blot analysis

Western blotting was performed following a standard protocol, which was previously described [53]. RBC-EV samples (~ 10 μg proteins) were solubilized with Laemmli sample buffer and were electrophoretically separated on 4–15% Criterion™ TGX Stain-Free™ Protein Gel (Bio-Rad Laboratories, USA) and then transferred to polyvinylidene difluoride (PVDF) membranes (Bio-Rad Laboratories, USA). The membrane was probed with corresponding primary antibodies (CD235a (MA5–12484, in a bovine serum albumin (BSA)-free PBS buffer, Thermo Fisher Scientific, USA); EAAT2 (MAB 2262; Millipore Sigma, USA); EAAT1 (A-3, sc-515,839, Santa Cruz Biotechnology, USA); anti-α-syn (clone 42, 624,096, BD Bioscience, USA)) overnight at 4 °C. After washing, membranes were then incubated with appropriate horseradish peroxidase (HRP)-conjugated secondary antibodies (Abcam, USA). The immunoreactive bands were visualized using ECL reagents (Amersham Pharmacia Biotech, Buckinghamshire, UK).

Dot blot analysis was performed following a standard protocol (Abcam, USA). Samples were spotted directly onto a nitrocellulose membrane. The membrane was then incubated with primary antibodies (MJF14–6–4-2 for oligomeric α-syn) overnight at 4 °C, followed by secondary horseradish peroxidase (HRP) conjugated antibodies (Abcam, USA) for 0.5 h at room temperature. Proteins were visualized using ECL reagents (Amersham Pharmacia Biotech, Buckinghamshire, UK).

### Statistical analysis

Statistical analysis was performed using Prism 8.0 (GraphPad, USA). The group differences were assessed by one-way ANOVA analysis (for three or more groups), Two-way ANOVA analysis (for three or more groups with two factors) or t-test (for two groups), followed by Tukey-Kramer’s post-hoc test, Sidak’s multiple comparisons post-test and Dunn’s post-hoc test, respectively, for multiple comparisons. Values with *p* < 0.05 were regarded as significant.

## Supplementary information

**Additional file 1 Supplemental Figure 1** Characterization of RBC-EV. (**a-d**) The size distribution (**a-c**) and total particle number (**d**) of cultured RBC-EVs and immunocaptured RBC EVs (anti-CD235a and IgG capture) were measured in each peak by NTA. (**e**) western blot analysis using lysate of anti-CD235a immunocaptured RBC EVs performed with antibodies against CD235a. (**f**) Representative CryoEM images of anti-CD235a immunocaptured RBC EVs co-labeled with immunogold against CD63 (Scale bar, 100 nm). **Supplemental Figure 2** Co-localization of KIR4.1 and MJFR14 (**a**) Representative images of human post mortem tissues (striatum (STR) and substantia nigra (SN)) co-labeled with KIR4.1 MJFR14 and GFAP. **Supplemental Figure 3** DiI labeled RBC-EVs are stable and EAAT1 does not form a complex with α-syn (**a**) Representative images of cultured astrocytes treated with DiI labeled RBC-EVs co-labeling with GFAP and 211. Note that DiI labeled RBC-EVs often co-localized with 211 positive signals. (**b**) Quantification analysis of percentage of astrocytes containing EAAT1/211 complexes. (**c**) Western blot analysis of EAAT1 (E1) and EAAT2 (E2) immunoprecipitates (IP) from the lysates of A53T mouse brain performed with antibodies against E1 or E2 and α-syn (211). IP with control nonimmune rabbit immunoglobulins (IgG) served as control. **Supplemental Figure 4** Co-localization of EAAT2 and MJFR14 (**a**) Representative images of human post mortem tissues (striatum (STR) and substantia nigra (SN)) co-labeled with EAAT2 and MJFR14. **Supplemental Table 1.** Characteristics of the clinical cohort of plasma samples. **Supplemental Table 2** Characteristics of the plasma pooling information**. Supplemental Table 3.** Characteristics of the clinical cohort of postmortem brain tissues.

## Data Availability

All the data included in this study are available and will be provided transparently upon request to the corresponding author.

## Abbreviations

α-syn: α-Synuclein
RBC: Red blood cell
EV: Extracellular vesicle
BBB: Blood–brain barrier
EAAT2: Excitatory amino acid transporter 2
WT: Wild type
LPS: Lipopolysaccharide
GFAP: Glial fibrillary acidic protein
PL: Proximity ligation

## References

[1] Ahn TB, Kim SY, Kim JY, Park SS, Lee DS, Min HJ, Kim YK, Kim SE, Kim JM, Kim HJ, Cho J, Jeon BS (2008). Alpha-Synuclein gene duplication is present in sporadic Parkinson disease. Neurology.

[2] Allen NJ, Eroglu C (2017). Cell biology of astrocyte-synapse interactions. Neuron.

[3] Almutairi MM, Gong C, Xu YG, Chang Y, Shi H (2016). Factors controlling permeability of the blood-brain barrier. Cell Mol Life Sci.

[4] Alvarez JI, Katayama T, Prat A (2013). Glial influence on the blood brain barrier. Glia.

[5] Ankarcrona M, Dypbukt JM, Bonfoco E, Zhivotovsky B, Orrenius S, Lipton SA, Nicotera P (1995). Glutamate-induced neuronal death: a succession of necrosis or apoptosis depending on mitochondrial function. Neuron.

[6] Banks WA, Kovac A, Majerova P, Bullock KM, Shi M, Zhang J (2017). Tau proteins cross the blood-brain barrier. J Alzheimers Dis.

[7] Barbour R, Kling K, Anderson JP, Banducci K, Cole T, Diep L, Fox M, Goldstein JM, Soriano F, Seubert P, Chilcote TJ (2008). Red blood cells are the major source of alpha-synuclein in blood. Neurodegener Dis.

[8] Blasberg RG, Fenstermacher JD, Patlak CS (1983). Transport of alpha-aminoisobutyric acid across brain capillary and cellular membranes. J Cereb Blood Flow Metab.

[9] Booth HDE, Hirst WD, Wade-Martins R (2017). The role of astrocyte dysfunction in Parkinson's disease pathogenesis. Trends Neurosci.

[10] Braak H, Del Tredici K (2017). Neuropathological staging of brain pathology in sporadic Parkinson's disease: separating the wheat from the chaff. J Park Dis.

[11] Braak H, Del Tredici K, Rub U, de Vos RA, Jansen Steur EN, Braak E (2003). Staging of brain pathology related to sporadic Parkinson's disease. Neurobiol Aging.

[12] Braak H, Sastre M, Del Tredici K (2007). Development of alpha-synuclein immunoreactive astrocytes in the forebrain parallels stages of intraneuronal pathology in sporadic Parkinson's disease. Acta Neuropathol.

[13] Bridi JC, Hirth F (2018). Mechanisms of alpha-Synuclein induced Synaptopathy in Parkinson's disease. Front Neurosci.

[14] Butchbach ME, Tian G, Guo H, Lin CL (2004). Association of excitatory amino acid transporters, especially EAAT2, with cholesterol-rich lipid raft microdomains: importance for excitatory amino acid transporter localization and function. J Biol Chem.

[15] Chavarria C, Rodriguez-Bottero S, Quijano C, Cassina P, Souza JM (2018). Impact of monomeric, oligomeric and fibrillar alpha-synuclein on astrocyte reactivity and toxicity to neurons. Biochem J.

[16] Cholerton BA, Zabetian CP, Quinn JF, Chung KA, Peterson A, Espay AJ, Revilla FJ, Devoto J, Watson GS, Hu SC, Edwards KL, Montine TJ, Leverenz JB (2013). Pacific Northwest Udall center of excellence clinical consortium: study design and baseline cohort characteristics. J Park Dis.

[17] Chung EK, Chen LW, Chan YS, Yung KK (2008). Downregulation of glial glutamate transporters after dopamine denervation in the striatum of 6-hydroxydopamine-lesioned rats. J Comp Neurol.

[18] Chung WS, Allen NJ, Eroglu C (2015). Astrocytes control synapse formation, function, and elimination. Cold Spring Harb Perspect Biol.

[19] Comi C, Magistrelli L, Oggioni GD, Carecchio M, Fleetwood T, Cantello R, Mancini F, Antonini A (2014). Peripheral nervous system involvement in Parkinson's disease: evidence and controversies. Parkinsonism Relat Disord.

[20] Cui Y, Yang Y, Ni Z, Dong Y, Cai G, Foncelle A, Ma S, Sang K, Tang S, Li Y, Shen Y, Berry H, Wu S, Hu H (2018). Astroglial Kir4.1 in the lateral habenula drives neuronal bursts in depression. Nature.

[21] da Fonseca AC, Matias D, Garcia C, Amaral R, Geraldo LH, Freitas C, Lima FR (2014). The impact of microglial activation on blood-brain barrier in brain diseases. Front Cell Neurosci.

[22] Derkinderen P, Rouaud T, Lebouvier T, Bruley des Varannes S, Neunlist M, De Giorgio R (2011). Parkinson disease: the enteric nervous system spills its guts. Neurology.

[23] Desai BS, Monahan AJ, Carvey PM, Hendey B (2007). Blood-brain barrier pathology in Alzheimer's and Parkinson's disease: implications for drug therapy. Cell Transplant.

[24] Dunn KW, Kamocka MM, McDonald JH (2011). A practical guide to evaluating colocalization in biological microscopy. Am J Physiol Cell Physiol.

[25] El-Agnaf OM, Salem SA, Paleologou KE, Curran MD, Gibson MJ, Court JA, Schlossmacher MG, Allsop D (2006). Detection of oligomeric forms of alpha-synuclein protein in human plasma as a potential biomarker for Parkinson's disease. FASEB J.

[26] Fazio P, Svenningsson P, Cselenyi Z, Halldin C, Farde L, Varrone A (2018). Nigrostriatal dopamine transporter availability in early Parkinson's disease. Mov Disord.

[27] Filippi L, Manni C, Pierantozzi M, Brusa L, Danieli R, Stanzione P, Schillaci O (2005). 123I-FP-CIT semi-quantitative SPECT detects preclinical bilateral dopaminergic deficit in early Parkinson's disease with unilateral symptoms. Nucl Med Commun.

[28] Giasson BI, Duda JE, Quinn SM, Zhang B, Trojanowski JQ, Lee VM (2002). Neuronal alpha-synucleinopathy with severe movement disorder in mice expressing A53T human alpha-synuclein. Neuron.

[29] Gray MT, Woulfe JM (2015). Striatal blood-brain barrier permeability in Parkinson's disease. J Cereb Blood Flow Metab.

[30] Gu XL, Long CX, Sun L, Xie C, Lin X, Cai H (2010). Astrocytic expression of Parkinson's disease-related A53T alpha-synuclein causes neurodegeneration in mice. Mol Brain.

[31] Guo H, Lai L, Butchbach ME, Stockinger MP, Shan X, Bishop GA, Lin CL (2003). Increased expression of the glial glutamate transporter EAAT2 modulates excitotoxicity and delays the onset but not the outcome of ALS in mice. Hum Mol Genet.

[32] Gustafsson G, Lindstrom V, Rostami J, Nordstrom E, Lannfelt L, Bergstrom J, Ingelsson M, Erlandsson A (2017). Alpha-synuclein oligomer-selective antibodies reduce intracellular accumulation and mitochondrial impairment in alpha-synuclein exposed astrocytes. J Neuroinflammation.

[33] Hoekstra JG, Cook TJ, Stewart T, Mattison H, Dreisbach MT, Hoffer ZS, Zhang J (2015). Astrocytic dynamin-like protein 1 regulates neuronal protection against excitotoxicity in Parkinson disease. Am J Pathol.

[34] Holmer HK, Keyghobadi M, Moore C, Meshul CK (2005). l-dopa-induced reversal in striatal glutamate following partial depletion of nigrostriatal dopamine with 1-methyl-4-phenyl-1,2,3,6-tetrahydropyridine. Neuroscience.

[35] Holmqvist S, Chutna O, Bousset L, Aldrin-Kirk P, Li W, Bjorklund T, Wang ZY, Roybon L, Melki R, Li JY (2014). Direct evidence of Parkinson pathology spread from the gastrointestinal tract to the brain in rats. Acta Neuropathol.

[36] Holmseth S, Scott HA, Real K, Lehre KP, Leergaard TB, Bjaalie JG, Danbolt NC (2009). The concentrations and distributions of three C-terminal variants of the GLT1 (EAAT2; slc1a2) glutamate transporter protein in rat brain tissue suggest differential regulation. Neuroscience.

[37] Hoogland ICM, Westhoff D, Engelen-Lee JY, Melief J, Valls Seron M, Houben-Weerts J, Huitinga I, van Westerloo DJ, van der Poll T, van Gool WA, van de Beek D (2018). Microglial activation after systemic stimulation with lipopolysaccharide and Escherichia coli. Front Cell Neurosci.

[38] Jain N, Bhasne K, Hemaswasthi M, Mukhopadhyay S (2013). Structural and dynamical insights into the membrane-bound alpha-synuclein. PLoS One.

[39] Janezic S, Threlfell S, Dodson PD, Dowie MJ, Taylor TN, Potgieter D, Parkkinen L, Senior SL, Anwar S, Ryan B, Deltheil T, Kosillo P, Cioroch M, Wagner K, Ansorge O, Bannerman DM, Bolam JP, Magill PJ, Cragg SJ, Wade-Martins R (2013). Deficits in dopaminergic transmission precede neuron loss and dysfunction in a new Parkinson model. Proc Natl Acad Sci U S A.

[40] Jankovic J (2008). Parkinson's disease: clinical features and diagnosis. J Neurol Neurosurg Psychiatry.

[41] Kim JM, Cha SH, Choi YR, Jou I, Joe EH, Park SM (2016). DJ-1 deficiency impairs glutamate uptake into astrocytes via the regulation of flotillin-1 and caveolin-1 expression. Sci Rep.

[42] Kortekaas R, Leenders KL, van Oostrom JC, Vaalburg W, Bart J, Willemsen AT, Hendrikse NH (2005). Blood-brain barrier dysfunction in parkinsonian midbrain in vivo. Ann Neurol.

[43] Lamontagne-Proulx J, St-Amour I, Labib R, Pilon J, Denis HL, Cloutier N, Roux-Dalvai F, Vincent AT, Mason SL, Williams-Gray C, Duchez AC, Droit A, Lacroix S, Dupre N, Langlois M, Chouinard S, Panisset M, Barker RA, Boilard E, Cicchetti F (2019). Portrait of blood-derived extracellular vesicles in patients with Parkinson's disease. Neurobiol Dis.

[44] Lassen LB, Gregersen E, Isager AK, Betzer C, Kofoed RH, Jensen PH (2018). ELISA method to detect alpha-synuclein oligomers in cell and animal models. PLoS One.

[45] Lauderback CM, Hackett JM, Huang FF, Keller JN, Szweda LI, Markesbery WR, Butterfield DA (2001). The glial glutamate transporter, GLT-1, is oxidatively modified by 4-hydroxy-2-nonenal in the Alzheimer's disease brain: the role of Abeta1-42. J Neurochem.

[46] Lee HJ, Suk JE, Patrick C, Bae EJ, Cho JH, Rho S, Hwang D, Masliah E, Lee SJ (2010). Direct transfer of alpha-synuclein from neuron to astroglia causes inflammatory responses in synucleinopathies. J Biol Chem.

[47] Lee SG, Su ZZ, Emdad L, Gupta P, Sarkar D, Borjabad A, Volsky DJ, Fisher PB (2008). Mechanism of ceftriaxone induction of excitatory amino acid transporter-2 expression and glutamate uptake in primary human astrocytes. J Biol Chem.

[48] Leshchyns'ka I, Liew HT, Shepherd C, Halliday GM, Stevens CH, Ke YD, Ittner LM, Sytnyk V (2015). Abeta-dependent reduction of NCAM2-mediated synaptic adhesion contributes to synapse loss in Alzheimer's disease. Nat Commun.

[49] Lewerenz J, Maher P (2015). Chronic glutamate toxicity in neurodegenerative diseases-what is the evidence?. Front Neurosci.

[50] Lin CL, Kong Q, Cuny GD, Glicksman MA (2012). Glutamate transporter EAAT2: a new target for the treatment of neurodegenerative diseases. Future Med Chem.

[51] Longhena F, Faustini G, Missale C, Pizzi M, Spano P, Bellucci A (2017). The contribution of alpha-Synuclein spreading to Parkinson's disease Synaptopathy. Neural Plast.

[52] Mathiisen TM, Lehre KP, Danbolt NC, Ottersen OP (2010). The perivascular astroglial sheath provides a complete covering of the brain microvessels: an electron microscopic 3D reconstruction. Glia.

[53] Matsumoto J, Stewart T, Sheng L, Li N, Bullock K, Song N, Shi M, Banks WA, Zhang J (2017). Transmission of alpha-synuclein-containing erythrocyte-derived extracellular vesicles across the blood-brain barrier via adsorptive mediated transcytosis: another mechanism for initiation and progression of Parkinson's disease?. Acta Neuropathol Commun.

[54] Mitosek-Szewczyk K, Sulkowski G, Stelmasiak Z, Struzynska L (2008). Expression of glutamate transporters GLT-1 and GLAST in different regions of rat brain during the course of experimental autoimmune encephalomyelitis. Neuroscience.

[55] Morales-Prieto DM, Stojiljkovic M, Diezel C, Streicher P-E, Röstel F, Lindner J, Weis S, Schmeer C, Marz M (2018) Peripheral blood exosomes pass blood-brain-barrier and induce glial cell activation. BioRxiv. 10.1101/471409

[56] Mori F, Tanji K, Yoshimoto M, Takahashi H, Wakabayashi K (2002). Demonstration of alpha-synuclein immunoreactivity in neuronal and glial cytoplasm in normal human brain tissue using proteinase K and formic acid pretreatment. Exp Neurol.

[57] Neildez-Nguyen TM, Wajcman H, Marden MC, Bensidhoum M, Moncollin V, Giarratana MC, Kobari L, Thierry D, Douay L (2002). Human erythroid cells produced ex vivo at large scale differentiate into red blood cells in vivo. Nat Biotechnol.

[58] Nielsen S, Nagelhus EA, Amiry-Moghaddam M, Bourque C, Agre P, Ottersen OP (1997). Specialized membrane domains for water transport in glial cells: high-resolution immunogold cytochemistry of aquaporin-4 in rat brain. J Neurosci.

[59] Norden DM, Fenn AM, Dugan A, Godbout JP (2014). TGFbeta produced by IL-10 redirected astrocytes attenuates microglial activation. Glia.

[60] Nwaobi SE, Cuddapah VA, Patterson KC, Randolph AC, Olsen ML (2016). The role of glial-specific Kir4.1 in normal and pathological states of the CNS. Acta Neuropathol.

[61] Okumura N, Tsuji K, Nakahata T (1992). Changes in cell surface antigen expressions during proliferation and differentiation of human erythroid progenitors. Blood.

[62] Pan-Montojo F, Anichtchik O, Dening Y, Knels L, Pursche S, Jung R, Jackson S, Gille G, Spillantini MG, Reichmann H, Funk RH (2010). Progression of Parkinson's disease pathology is reproduced by intragastric administration of rotenone in mice. PLoS One.

[63] Pan-Montojo F, Schwarz M, Winkler C, Arnhold M, O'Sullivan GA, Pal A, Said J, Marsico G, Verbavatz JM, Rodrigo-Angulo M, Gille G, Funk RH, Reichmann H (2012). Environmental toxins trigger PD-like progression via increased alpha-synuclein release from enteric neurons in mice. Sci Rep.

[64] Papagiannakis N, Koros C, Stamelou M, Simitsi AM, Maniati M, Antonelou R, Papadimitriou D, Dermentzaki G, Moraitou M, Michelakakis H, Stefanis L (2018). Alpha-synuclein dimerization in erythrocytes of patients with genetic and non-genetic forms of Parkinson's disease. Neurosci Lett.

[65] Passmore LA, Russo CJ (2016). Specimen preparation for high-resolution Cryo-EM. Methods Enzymol.

[66] Peelaerts W, Bousset L, Van der Perren A, Moskalyuk A, Pulizzi R, Giugliano M, Van den Haute C, Melki R, Baekelandt V (2015). Alpha-Synuclein strains cause distinct synucleinopathies after local and systemic administration. Nature.

[67] Peng C, Gathagan RJ, Covell DJ, Medellin C, Stieber A, Robinson JL, Zhang B, Pitkin RM, Olufemi MF, Luk KC, Trojanowski JQ, Lee VM (2018). Cellular milieu imparts distinct pathological alpha-synuclein strains in alpha-synucleinopathies. Nature.

[68] Petzold GC, Albeanu DF, Sato TF, Murthy VN (2008). Coupling of neural activity to blood flow in olfactory glomeruli is mediated by astrocytic pathways. Neuron.

[69] Pretorius E, Swanepoel AC, Buys AV, Vermeulen N, Duim W, Kell DB (2014). Eryptosis as a marker of Parkinson's disease. Aging.

[70] Readnower RD, Chavko M, Adeeb S, Conroy MD, Pauly JR, McCarron RM, Sullivan PG (2010). Increase in blood-brain barrier permeability, oxidative stress, and activated microglia in a rat model of blast-induced traumatic brain injury. J Neurosci Res.

[71] Recasens A, Dehay B (2014). Alpha-synuclein spreading in Parkinson's disease. Front Neuroanat.

[72] Rey NL, Petit GH, Bousset L, Melki R, Brundin P (2013). Transfer of human alpha-synuclein from the olfactory bulb to interconnected brain regions in mice. Acta Neuropathol.

[73] Reynolds NP, Soragni A, Rabe M, Verdes D, Liverani E, Handschin S, Riek R, Seeger S (2011). Mechanism of membrane interaction and disruption by alpha-synuclein. J Am Chem Soc.

[74] Rostami J, Holmqvist S, Lindstrom V, Sigvardson J, Westermark GT, Ingelsson M, Bergstrom J, Roybon L, Erlandsson A (2017). Human astrocytes transfer aggregated alpha-Synuclein via tunneling nanotubes. J Neurosci.

[75] Sacino AN, Brooks M, Thomas MA, McKinney AB, Lee S, Regenhardt RW, McGarvey NH, Ayers JI, Notterpek L, Borchelt DR, Golde TE, Giasson BI (2014). Intramuscular injection of alpha-synuclein induces CNS alpha-synuclein pathology and a rapid-onset motor phenotype in transgenic mice. Proc Natl Acad Sci U S A.

[76] Schindelin J, Arganda-Carreras I, Frise E, Kaynig V, Longair M, Pietzsch T, Preibisch S, Rueden C, Saalfeld S, Schmid B, Tinevez JY, White DJ, Hartenstein V, Eliceiri K, Tomancak P, Cardona A (2012). Fiji: an open-source platform for biological-image analysis. Nat Methods.

[77] Sheng L, Leshchyns'ka I, Sytnyk V (2015). Neural cell adhesion molecule 2 promotes the formation of filopodia and neurite branching by inducing submembrane increases in Ca2+ levels. J Neurosci.

[78] Sheng L, Leshchyns'ka I, Sytnyk V (2019). Neural cell adhesion molecule 2 (NCAM2)-induced c-Src-dependent propagation of submembrane Ca2+ spikes along dendrites inhibits synapse maturation. Cereb Cortex.

[79] Shi M, Kovac A, Korff A, Cook TJ, Ginghina C, Bullock KM, Yang L, Stewart T, Zheng D, Aro P, Atik A, Kerr KF, Zabetian CP, Peskind ER, Hu SC, Quinn JF, Galasko DR, Montine TJ, Banks WA, Zhang J (2016). CNS tau efflux via exosomes is likely increased in Parkinson's disease but not in Alzheimer's disease. Alzheimers Dement.

[80] Shi M, Liu C, Cook TJ, Bullock KM, Zhao Y, Ginghina C, Li Y, Aro P, Dator R, He C, Hipp MJ, Zabetian CP, Peskind ER, Hu SC, Quinn JF, Galasko DR, Banks WA, Zhang J (2014). Plasma exosomal alpha-synuclein is likely CNS-derived and increased in Parkinson's disease. Acta Neuropathol.

[81] Shi M, Zabetian CP, Hancock AM, Ginghina C, Hong Z, Yearout D, Chung KA, Quinn JF, Peskind ER, Galasko D, Jankovic J, Leverenz JB, Zhang J (2010). Significance and confounders of peripheral DJ-1 and alpha-synuclein in Parkinson's disease. Neurosci Lett.

[82] Shulman JM, De Jager PL, Feany MB (2011). Parkinson's disease: genetics and pathogenesis. Annu Rev Pathol.

[83] Sielewicz M, Scholz J, Hanslik L (1989). A five year follow-up of 605 cases of the MCCL (metal-cancellous cementless Lubeck) total hip prosthesis. Ital J Orthop Traumatol.

[84] Simard M, Arcuino G, Takano T, Liu QS, Nedergaard M (2003). Signaling at the gliovascular interface. J Neurosci.

[85] Singh B, Covelo A, Martell-Martinez H, Nanclares C, Sherman MA, Okematti E, Meints J, Teravskis PJ, Gallardo C, Savonenko AV, Benneyworth MA, Lesne SE, Liao D, Araque A, Lee MK (2019). Tau is required for progressive synaptic and memory deficits in a transgenic mouse model of alpha-synucleinopathy. Acta Neuropathol.

[86] Sofroniew MV, Vinters HV (2010). Astrocytes: biology and pathology. Acta Neuropathol.

[87] Sorrentino ZA, Giasson BI, Chakrabarty P (2019). Alpha-Synuclein and astrocytes: tracing the pathways from homeostasis to neurodegeneration in Lewy body disease. Acta Neuropathol.

[88] Stuendl A, Kunadt M, Kruse N, Bartels C, Moebius W, Danzer KM, Mollenhauer B, Schneider A (2016). Induction of alpha-synuclein aggregate formation by CSF exosomes from patients with Parkinson's disease and dementia with Lewy bodies. Brain.

[89] Sui YT, Bullock KM, Erickson MA, Zhang J, Banks WA (2014). Alpha synuclein is transported into and out of the brain by the blood-brain barrier. Peptides.

[90] Suloway C, Pulokas J, Fellmann D, Cheng A, Guerra F, Quispe J, Stagg S, Potter CS, Carragher B (2005). Automated molecular microscopy: the new Leginon system. J Struct Biol.

[91] Sweeney MD, Sagare AP, Zlokovic BV (2018). Blood-brain barrier breakdown in Alzheimer disease and other neurodegenerative disorders. Nat Rev Neurol.

[92] Sweeney MD, Zhao Z, Montagne A, Nelson AR, Zlokovic BV (2019). Blood-brain barrier: from physiology to disease and Back. Physiol Rev.

[93] Teravskis PJ, Covelo A, Miller EC, Singh B, Martell-Martinez HA, Benneyworth MA, Gallardo C, Oxnard BR, Araque A, Lee MK, Liao D (2018). A53T mutant alpha-Synuclein induces tau-dependent postsynaptic impairment independently of neurodegenerative changes. J Neurosci.

[94] Tian C, Liu G, Gao L, Soltys D, Pan C, Stewart T, Shi M, Xie Z, Liu N, Feng T, Zhang J (2019). Erythrocytic alpha-Synuclein as a potential biomarker for Parkinson's disease. Transl Neurodegeneration.

[95] Tian G, Kong Q, Lai L, Ray-Chaudhury A, Lin CL (2010). Increased expression of cholesterol 24S-hydroxylase results in disruption of glial glutamate transporter EAAT2 association with lipid rafts: a potential role in Alzheimer's disease. J Neurochem.

[96] Tissingh G, Booij J, Bergmans P, Winogrodzka A, Janssen AG, van Royen EA, Stoof JC, Wolters EC (1998). Iodine-123-N-omega-fluoropropyl-2beta-carbomethoxy-3beta-(4-iod ophenyl) tropane SPECT in healthy controls and early-stage, drug-naive Parkinson's disease. J Nucl Med.

[97] Turski L, Bressler K, Rettig KJ, Loschmann PA, Wachtel H (1991). Protection of substantia nigra from MPP+ neurotoxicity by N-methyl-D-aspartate antagonists. Nature.

[98] Ulusoy A, Rusconi R, Perez-Revuelta BI, Musgrove RE, Helwig M, Winzen-Reichert B, Di Monte DA (2013). Caudo-rostral brain spreading of alpha-synuclein through vagal connections. EMBO Mol Med.

[99] Vicente Miranda H, Cassio R, Correia-Guedes L, Gomes MA, Chegao A, Miranda E, Soares T, Coelho M, Rosa MM, Ferreira JJ, Outeiro TF (2017). Posttranslational modifications of blood-derived alpha-synuclein as biochemical markers for Parkinson's disease. Sci Rep.

[100] Wakabayashi K, Hayashi S, Yoshimoto M, Kudo H, Takahashi H (2000). NACP/alpha-synuclein-positive filamentous inclusions in astrocytes and oligodendrocytes of Parkinson's disease brains. Acta Neuropathol.

[101] Wang S, Chu CH, Stewart T, Ginghina C, Wang Y, Nie H, Guo M, Wilson B, Hong JS, Zhang J (2015). Alpha-Synuclein, a chemoattractant, directs microglial migration via H2O2-dependent Lyn phosphorylation. Proc Natl Acad Sci U S A.

[102] Wong YC, Krainc D (2017). Alpha-synuclein toxicity in neurodegeneration: mechanism and therapeutic strategies. Nat Med.

[103] Zagrean AM, Hermann DM, Opris I, Zagrean L, Popa-Wagner A (2018). Multicellular crosstalk between Exosomes and the neurovascular unit after cerebral ischemia. Therapeut Implications Front Neurosci.

[104] Zhao HQ, Li FF, Wang Z, Wang XM, Feng T (2016). A comparative study of the amount of alpha-synuclein in ischemic stroke and Parkinson's disease. Neurol Sci.

[105] Zuddas A, Oberto G, Vaglini F, Fascetti F, Fornai F, Corsini GU (1992). MK-801 prevents 1-methyl-4-phenyl-1,2,3,6-tetrahydropyridine-induced parkinsonism in primates. J Neurochem.

