# Supplementary material for: Erythrocytic α-synuclein contained in microvesicles regulates astrocytic glutamate homeostasis: a new perspective on Parkinson’s disease pathogenesis
Source: Acta Neuropathol Commun. 2020 Jul 8;8:102. doi: 10.1186/s40478-020-00983-w (PMC7346449; doi:10.1186/s40478-020-00983-w)
Supplement: Supplementary file 1 — Additional file 1 Supplemental Figure 1 Characterization of RBC-EV. (a-d) The size distribution (a-c) and total particle number (d) of cultured RBC-EVs and immunocaptured RBC EVs (anti-CD235a and IgG capture) were measured in each peak by NTA. (e) western blot analysis using lysate of anti-CD235a immunocaptured RBC EVs performed with antibodies against CD235a. (f) Representative CryoEM images of anti-CD235a immunocaptured RBC EVs co-labeled with immunogold against CD63 (Scale bar, 100 nm). Supplemental Figure 2 Co-localization of KIR4.1 and MJFR14 (a) Representative images of human post mortem tissues (striatum (STR) and substantia nigra (SN)) co-labeled with KIR4.1 MJFR14 and GFAP. Supplemental Figure 3 DiI labeled RBC-EVs are stable and EAAT1 does not form a complex with α-syn (a) Representative images of cultured astrocytes treated with DiI labeled RBC-EVs co-labeling with GFAP and 211. Note that DiI labeled RBC-EVs often co-localized with 211 positive signals. (b) Quantification analysis of percentage of astrocytes containing EAAT1/211 complexes. (c) Western blot analysis of EAAT1 (E1) and EAAT2 (E2) immunoprecipitates (IP) from the lysates of A53T mouse brain performed with antibodies against E1 or E2 and α-syn (211). IP with control nonimmune rabbit immunoglobulins (IgG) served as control. Supplemental Figure 4 Co-localization of EAAT2 and MJFR14 (a) Representative images of human post mortem tissues (striatum (STR) and substantia nigra (SN)) co-labeled with EAAT2 and MJFR14. Supplemental Table 1. Characteristics of the clinical cohort of plasma samples. Supplemental Table 2 Characteristics of the plasma pooling information. Supplemental Table 3. Characteristics of the clinical cohort of postmortem brain tissues. [file 40478_2020_983_MOESM1_ESM.docx]

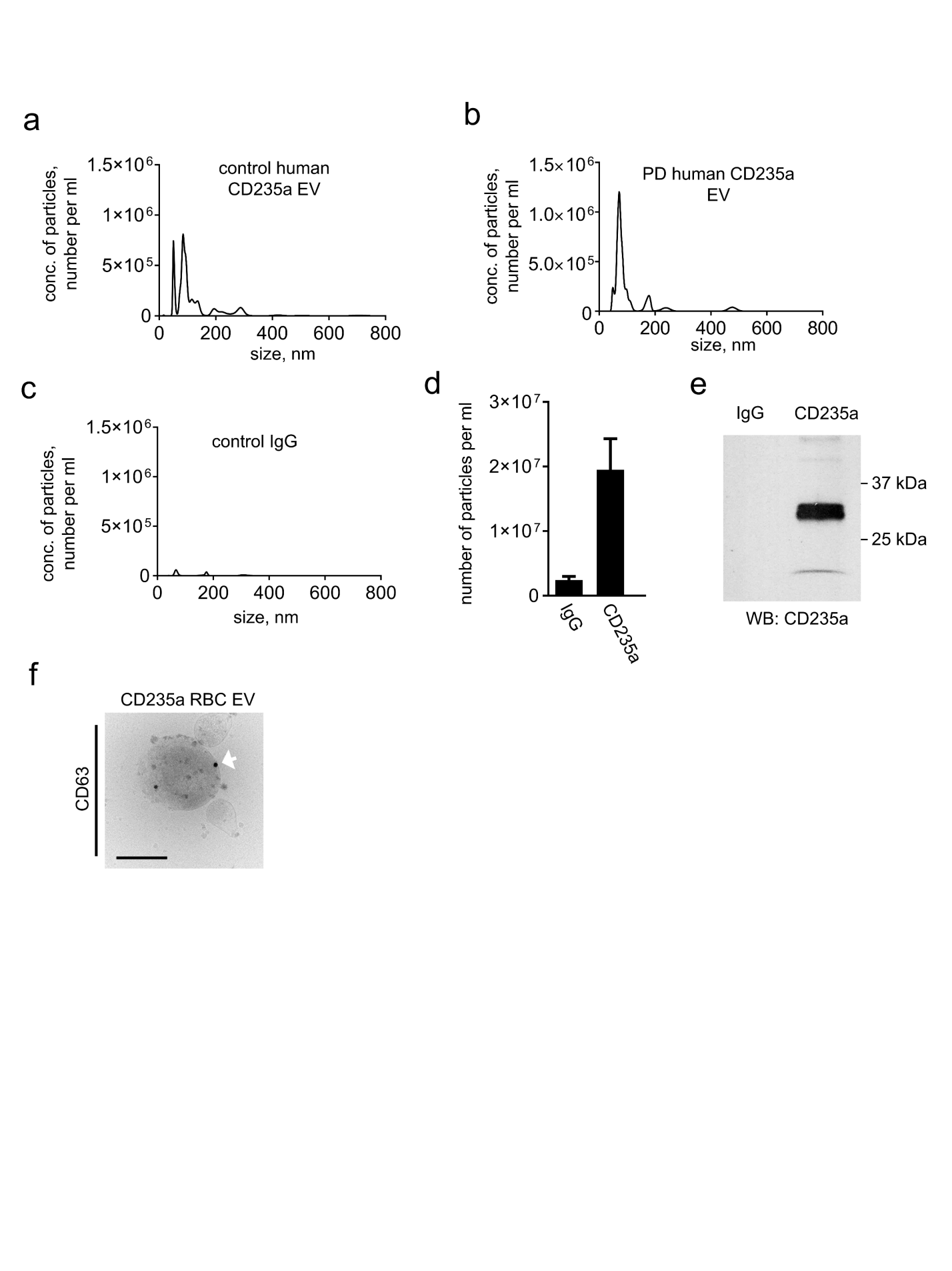
**Supplemental data**

**Supplemental Figure 1 Characterization of RBC-EV**

(**a-d**) The size distribution (**a-c**) and total particle number (**d**) of cultured RBC-EVs and immunocaptured RBC EVs (anti-CD235a and IgG capture) were measured in each peak by NTA. (**e**) western blot analysis using lysate of anti-CD235a immunocaptured RBC EVs performed with antibodies against CD235a. (**f**) Representative CryoEM images of anti-CD235a immunocaptured RBC EVs co-labeled with immunogold against CD63 (Scale bar, 100 nm).


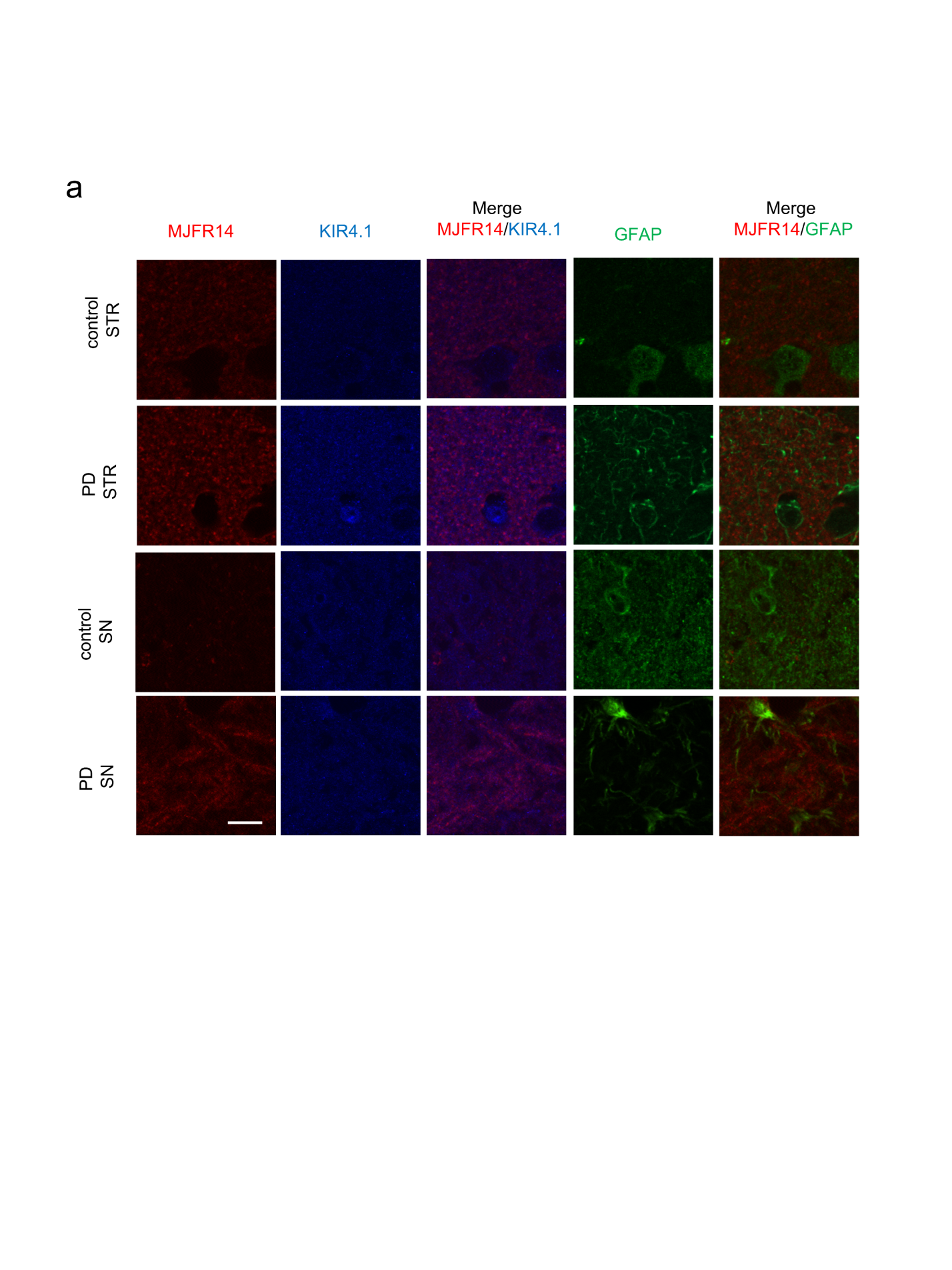


**Supplemental Figure 2 Co-localization of KIR4.1 and MJFR14**

(**a**) Representative images of human post mortem tissues (striatum (STR) and substantia nigra (SN)) co-labeled with KIR4.1 MJFR14 and GFAP.


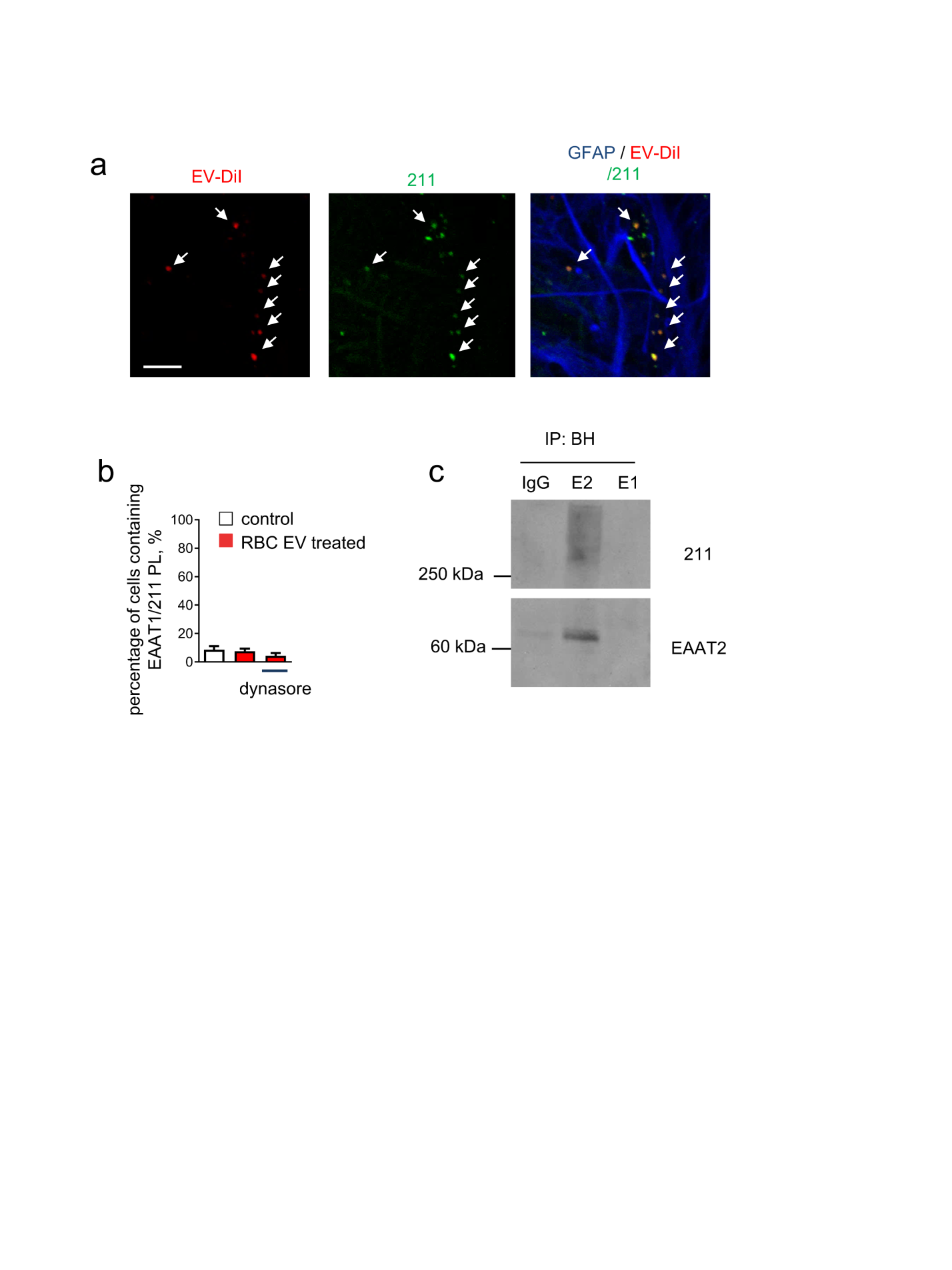


**Supplemental Figure 3 DiI labeled RBC-EVs are stable and EAAT1 does not form a complex with α-syn**

(**a**) Representative images of cultured astrocytes treated with DiI labeled RBC-EVs co-labeling with GFAP and 211. Note that DiI labeled RBC-EVs often co-localized with 211 positive signals. (**b**) Quantification analysis of percentage of astrocytes containing EAAT1/211 complexes. (**c**) Western blot analysis of EAAT1 (E1) and EAAT2 (E2) immunoprecipitates (IP) from the lysates of A53T mouse brain performed with antibodies against E1 or E2 and α-syn (211). IP with control nonimmune rabbit immunoglobulins (IgG) served as control.


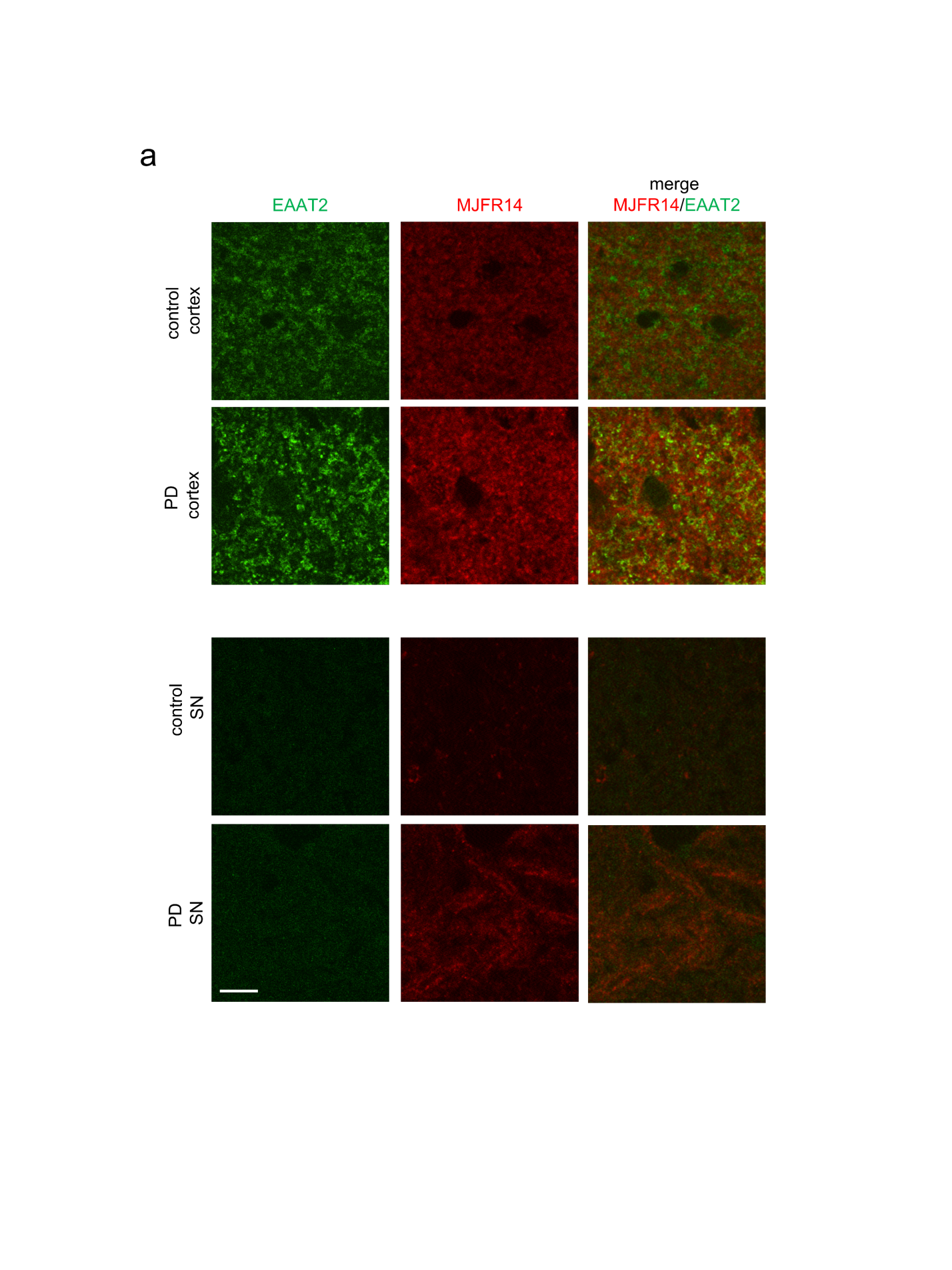


**Supplemental Figure 4 Co-localization of EAAT2 and MJFR14**

(**a**) Representative images of human post mortem tissues (striatum (STR) and substantia nigra (SN)) co-labeled with EAAT2 and MJFR14.

**Supplemental Table 1.** Characteristics of the clinical cohort of plasma samples

| Characteristic | Healthy Control | Parkinson’s Disease |
| --- | --- | --- |
| Number | 59 | 109 |
| Sex (Female/Male) | 26/33 | 29/90 |
| Age (mean ± SD) | 65.85 ± 0.89 | 69.86 ± 0.92 |
| MDS-UPDRS III (mean ± SD) | n/a | 10 ± 0.77 |

**Supplemental Table 2** Characteristics of the plasma pooling information

| Sample | Number of cases | Age  (mean ± SD) | Sex  (Female/Male) | UPDRS |
| --- | --- | --- | --- | --- |
| Control |  |  |  |  |
| pool1 | 6 | 71.50 ± 1.89 | 3/3 |  |
| pool2 | 6 | 70.66 ± 1.02 | 3/3 |  |
| pool3 | 6 | 69.50 ± 3.12 | 3/3 |  |
| pool4 | 6 | 72.50 ± 1.72 | 3/3 |  |
| pool5 | 5 | 61.60 ± 6.77 | 1/4 |  |
| pool6 | 4 | 62.75 ± 2.56 | 1/3 |  |
| pool7 | 6 | 71.00 ± 3.59 | 3/3 |  |
| pool8 | 5 | 70.46 ± 1.20 | 3/2 |  |
| pool9 | 4 | 69.75 ± 2.29 | 2/2 |  |
| pool10 | 4 | 71.40 ± 3.32 | 3/1 |  |
| pool11 | 4 | 74.60 ± 0.48 | 2/2 |  |
| pool12 | 3 | 71.33 ± 1.85 | 2/1 |  |
| Parkinson’s Disease |  |  |  |  |
| pool1 | 10 | 66.55 ± 3.88 | 2/8 | 10 ± 2.14 |
| pool2 | 10 | 63.53 ± 3.93 | 3/7 | 11 ± 2.50 |
| pool3 | 10 | 64.62 ± 3.30 | 3/7 | 8 ± 3.21 |
| pool4 | 10 | 65.73 ± 2.69 | 3/7 | 10 ± 3.21 |
| pool5 | 10 | 66.05 ± 2.65 | 3/7 | 11 ± 2.45 |
| pool6 | 10 | 66.27 ± 2.62 | 3/7 | 10 ± 1.91 |
| pool7 | 10 | 66.54 ± 2.54 | 3/7 | 12 ± 4.31 |
| pool8 | 10 | 63.04 ± 3.10 | 4/6 | 11 ± 1.97 |
| pool9 | 9 | 66.14 ± 2.70 | 2/7 | 9 ± 1.60 |
| pool10 | 9 | 68.86 ± 3.23 | 1/8 | 17 ± 1.64 |
| pool11 | 10 | 67.94 ± 2.99 | 2/8 | 8 ± 2.14 |

**Supplemental Table 3.** Characteristics of the clinical cohort of postmortem brain tissues

| Characteristic | Healthy Control | | PD | |
| --- | --- | --- | --- | --- |
| Number | 5 | | 5 |  |
| Sex (Female/Male) | 2/3 | | 2/3 |  |
| Age (mean ± SD) | 81 ± 2.17 | | 84.6 ± 2.98 |  |
| PMI mean hrs (range) | 25.138 (6-67.25) | | 10.716 (3-28.75) |  |
| BraakStage | I (1); II (4) | | V (3); VI (1); N/A (1) |  |
| Lewy Body Stage | N/A | Neocortical (diffuse) (2)  Brainstem Predominant (3) | |  |
|  |  | |  | |
